# Sexual Dimorphism and Intraspecific Toxin Variation in *Crotalus durissus terrificus* Venom: Insights from Proteomics, Peptidomics, and Structural Modeling

**DOI:** 10.1021/acs.jproteome.6c00275

**Published:** 2026-09-04

**Authors:** Jackson G. Miyamoto, Jackelinne Y. Hayashi, Giuseppe G. Figueirêdo Leite, Sávio S. Sant’Anna, Hang Fai Kwok, Richard H. Valente, Anita M. Tanaka-Azevedo, Alexandre K. Tashima

**Affiliations:** † Department of Biochemistry, Escola Paulista de Medicina, 28105 Universidade Federal de São Paulo, São Paulo 04039-032, Brazil; ‡ Laboratory of Mass Spectrometry Applied to Biomolecules, Escola Paulista de Medicina, Universidade Federal de São Paulo, São Paulo 04039-032, Brazil; § Division of Infectious Diseases, Department of Medicine, Escola Paulista de Medicina, Universidade Federal de São Paulo, São Paulo 04039-032, Brazil; ∥ Laboratory of Herpetology, Butantan Institute, São Paulo 05503-900, Brazil; ⊥ Department of Biomedical Sciences, Faculty of Health Sciences, Avenida de Universidade, University of Macau, Taipa 999078, Macau, SAR; # Laboratory of Toxinology, Oswaldo Cruz Institute, 37903 Oswaldo Cruz Foundation, FIOCRUZ, Rio de Janeiro 21040-900, Brazil

**Keywords:** *Crotalus durissus terrificus*, snake venom, proteomics, peptidomics, mutation, sexual dimorphism

## Abstract

The venom of the South American rattlesnake *Crotalus durissus terrificus* (Cdt) is renowned for its minimalist yet highly toxic arsenal. Intraspecific variability in expressed proteoforms and toxin content has been previously observed in Cdt venom, resulting in heterogeneous biological activities. In light of this, our study conducted an in-depth mass spectrometry-based analysis, combined with structural prediction to comprehensively assess Cdt venom variability and sexual dimorphism. Using peptidomic analysis, we identified 76 native peptides within Cdt venom, predominantly (63%) associated with noncanonical processing of crotoxin subunits, and 9 novel peptides via *de novo* sequencing. Quantitative analysis showed increased abundance of two crotapotin-derived peptides in females, while three novel peptides were significantly increased in males. Through quantitative proteomics, we characterized a repertoire of 33 proteins, with over one-third displaying sex-based differences in abundance. Furthermore, variant analysis revealed 12 novel toxin variants, generally at lower abundance than previously described proteoforms. Predictive three-dimensional structure modeling revealed conserved scaffolds, suggesting preservation of their biological activities. Addressing venom variability provides valuable insights into the intricate variability of Cdt venom, contributing to the understanding of its biological, medical, and biotechnological implications.

## Graphical abstract

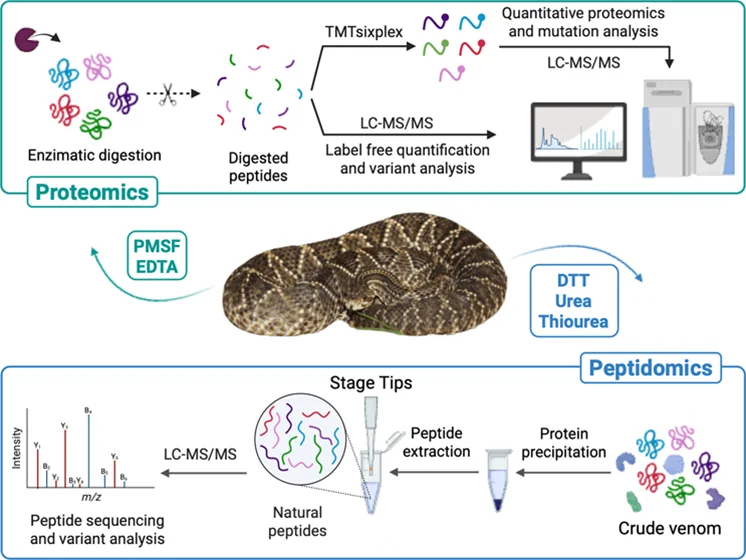

## Introduction

Snake venoms exhibit remarkable variability, reflecting evolutionary adaptation to diverse ecological niches, resulting in a wide spectrum of actions on prey and a range of pathological manifestations in human victims. This phenomenon unfolds through the evolution of novel venom compositions that overcome prey defenses, consequently exerting reciprocal selective pressure on prey resistance, shaping a coevolutionary arms race.^1^

Numerous mechanisms have been proposed to explain the diversity of toxin-coding families, encompassing gene duplication, domain loss, variation in expression levels, accelerated evolution under positive selection, and transcriptional and post-translational modifications.^2^ This reveals widespread accelerated evolution across multiple toxin isozyme families, with nucleotide substitutions exhibiting a pronounced preference for toxin-coding exons as opposed to nonvenomous genes’ introns and untranslated regions.^3,4^ Particularly, highly abundant proteins experience stronger positive selection and relaxed selective constraints, leading to the accumulation of mutations and the expansion of the phenotypic space.^5^

Contrary to the prevailing notion of single-gene optimization, the genome’s profusion of structural and functional paralogs confers a distinct selective advantage, particularly synergistic interactions.^6^ Rather than the dominant role of directional positive selection, where alleles with high fitness become fixed, evidence supports balancing selection in maintaining allelic diversity, potentially driven by heterozygote advantage.^7,8^

The venom of the South American rattlesnake species *Crotalus durissus terrificus* (Cdt) exhibits notable intraspecific variation in expressed toxins, toxin abundances, enzymatic activities, and toxicity.^9–15^ This variation arises from factors such as snake ontogeny, sex, geographic distribution, and captivity conditions.^13–16^ Electrophoresis and proteomics have shown clear differences between crotamine-positive and crotamine-negative *Crotalus durissus* venom pools, such as exclusive protein bands and quantitative differences in PLA_2_, SVSP, and LAAO levels between the two venom groups.^13^ Further chromatographic analyses of 315 Cdt individuals^14^ unveiled a more extensive array of proteins and peptides than the currently characterized toxin repertoire within Cdt venom. Rather than representing arbitrary divergences, individual compositional diversity appears to be a within-specimen paradigm.^15^

Understanding the functional variation within snake venom and unraveling the molecular mechanisms that underlie this phenomenon are essential for developing strategies to address the burden of one of the most neglected tropical diseases.^17,18^ In this regard, mass spectrometry has been playing a crucial role in exploring venom toxins, providing invaluable insights into interindividual and sexual variation,^19,20^ enabling the characterization of toxin proteoforms^21^ and thus, allowing a deeper comprehension of snake venom diversity.^22^ Here, we delve into the intricate complexities of snake venom diversity by exploring the compositional dynamics of Cdt venom toxins at the individual level. Through a combination of mass spectrometry-based strategies, this study used peptidomics, *de novo* analysis and label-free and TMT-labeling quantitative proteomics to characterize the individual composition of Cdt venom thoroughly. Specifically, we investigated sexual dimorphism, mapped variants within homologous sequences and analyzed the impact of amino acid variation on predicted three-dimensional structures and toxin stability.

## Materials and Methods

### Reagents

Proteolytic enzymes were purchased from Promega. Dithiothreitol and iodoacetamide were obtained from GE Life Sciences. Unless specified, all other reagents were obtained from Sigma-Aldrich.

### Venom Extraction and Ethical Approval

The research protocols and animal care guidelines adopted were followed in accordance with the standardization of the LH-IBu Protocol 1296/16 and approved by the Ethics Committees on the use of animals of Instituto Butantan and the Federal University of São Paulo (CEUAIB: 7967310720, CEUA n° 2589220322).^23^ The specimens used in this study were registered in the Brazilian National System for Management of Genetic Heritage and Associated Traditional Knowledge (SisGen: registration number A7DC273).^23^ Venom extractions were routinely performed every 60 days, and snakes were fed with rodents every 30 days, 7 days after extraction.^24^ Five female and five male specimens were selected for this study. All specimens had been maintained in captivity for at least three years at the time of venom extraction. Biometric data and geographic background from the specimens used for venom extraction are shown in . Venoms (200 μL) were collected into individual beakers kept in an ice bath and immediately aliquoted with proteinase inhibitors or denaturing solution, for different processing protocols (described in further detail below), followed by centrifugation at 1,700*g* at 4 °C for 20 min for debris removal.

### Sampling Localities and Map Generation

The collection localities of the ten *Crotalus durissus terrificus* specimens^23^ (Figure 1) were plotted from the coordinates of their municipal seats (IBGE, WGS84), with a small offset applied to colocated specimens to prevent marker overlap. The map was generated in R version 4.5.2 using the *geobr*, *sf*, *terra*, *elevatr*, *tidyterra*, *ggplot2*, *ggspatial*, and *ggrepel* packages. Administrative boundaries and the national biome delimitation were obtained from IBGE through *geobr*, and a shaded-relief base was derived from an AWS Terrain Tiles digital elevation model (SRTM-derived, public domain) via *elevatr*. The complete script is in the Supplementary R Script.

**Figure 1:**
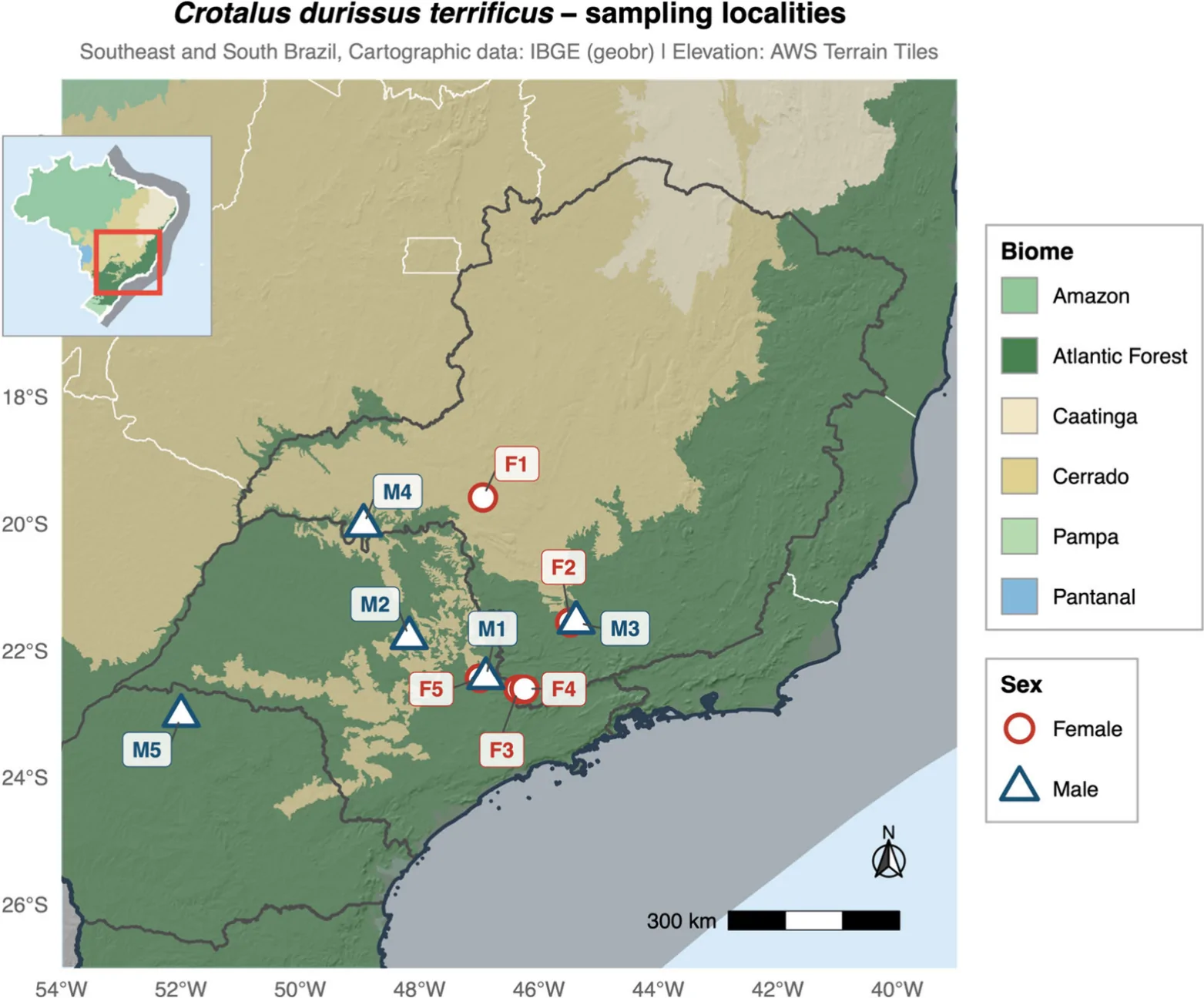
Geographic distribution of *Crotalus durissus terrificus* specimens used in this study. Sampling localities of five female (red circles) and five male (blue triangles) specimens are shown across the states of São Paulo, Minas Gerais, and Paraná (Southeast and South Brazil). Background colors indicate the Brazilian biomes (IBGE), overlaid on shaded relief derived from a digital elevation model (SRTM/NASA; Mapzen/AWS Terrain Tiles). The inset shows the location of the mapped region within Brazil. Cartographic data were obtained with the geobr R package.

### Venom Processing

To obtain the peptide fraction,^25^ 100 μL of crude venom was diluted (1:2) in a denaturing solution composed of 7 M urea, 2 M thiourea and 20 mM dithiothreitol (DTT). After centrifugation for debris removal, the solution was added to 4 mL of ice-cold acetone, stirred for 1 h at 4 °C, and then centrifuged at 19,000*g* and 4 °C for 15 min. The precipitate was resuspended in 2 mL of 80% acetonitrile (ACN) solution containing 2 mM hydrochloric acid, stirred for 2 h, and centrifuged under the same conditions. The supernatants of the acetone and ACN solutions were collected, vacuum-dried using a Concentrator Plus (Eppendorf) and stored at −80 °C.^23^

Venom samples for proteomic analysis were collected using a combination of serine protease and metalloproteinase inhibitors^26^ composed of ethylenediaminetetraacetic acid (EDTA) and phenylmethylsulfonyl fluoride (PMSF) as previously described at 5 mM and 2 mM, respectively.^26^ After debris removal, the supernatants were lyophilized and stored at −80 °C.

### Protein and Peptide Quantification

Protein and peptide concentrations were determined using the Bradford and Pierce Colorimetric Peptide assays (Thermo Scientific), respectively, in a Synergy HT microplate reader (BioTek). Following TMT labeling, peptide concentrations were measured at 280 nm with a NanoDrop spectrophotometer (Thermo Scientific) to normalize LC-MS/MS injection volumes.^23^

### Polyacrylamide Gel Electrophoresis (SDS-PAGE)

Individual venom samples were dissolved (1:2) in gel loading buffer (0.5 M Tris, 0.71 M 2-mercaptoethanol, 10% sodium dodecyl sulfate (SDS), 0.5% bromophenol blue, and 25% glycerol) and heated for 4 min at 95 °C for protein denaturation. Proteins were separated on 15% polyacrylamide gels by electrophoresis (PAGE) at 100 V for 120 min.^23^ For protein visualization, the gels were stained with a solution of 0.2% Coomassie R-250 (Bio-Rad), 45% methanol, and 10% acetic acid. Gels were scanned and measurements were performed using ImageJ software (version 2.9.0).

### Enzymatic Digestion of Proteins and Peptide Tagging

Individual venom protein samples were solubilized in 50 mM ammonium bicarbonate buffer and incubated with 0.1% Rapigest surfactant (Waters) at 80 °C for 15 min. The samples (100 μg aliquots) were reduced with DTT to a final concentration of 5 mM at 60 °C for 30 min, then alkylated with 15 mM iodoacetamide in the dark at room temperature for 30 min. Enzymatic digestions were carried out using rLys-C (for DIA and label-free quantification) or a trypsin/Lys-C mix (DDA and TMT quantification) at 37 °C overnight, according to the manufacturer’s recommendations.^23^ Trifluoroacetic acid (TFA) was added to a final concentration of 0.7% to stop digestion and hydrolyze the surfactant.

For isobaric labeling, peptides were resuspended in 50 mM triethylammonium bicarbonate and labeled with TMTsixplex reagents (Thermo Scientific) according to the manufacturer’s recommendations with minor modifications.^23,27^ A pooled global internal standard (GIS) was assigned to channel 126, whereas individual samples were distributed across channels 127–131. Following labeling, samples were combined in equal proportions, vacuum-dried, and stored at −80 °C until LC-MS/MS analysis.

### Sample Desalting and Fractionation

Peptide extracts and proteolytic digests were desalted using C18 StageTips^28^ assembled with InertSep RP-C18 (GL Sciences) and SDB-XC (Empore, 3M) membranes. Peptides were eluted with 40% ACN containing 0.1% TFA.^23^

### Mass Spectrometry Workflows

Three complementary LC-MS/MS workflows were employed.^23^ DIA acquisition mode (MS^E^) on a SYNAPT G2 mass spectrometer (Waters) was used for native peptidomics, while both MS^E^ and HDMS^E^ modes on the same platform were used for label-free proteomics. The DIA data set was additionally searched for sequence variants using the SPIDER module of PEAKS Studio. Individual-level quantitative comparison between female and male specimens was based on TMT isobaric labeling coupled to DDA-MS/MS on a Q Exactive HF-X Orbitrap instrument (Thermo Scientific). Methodological details are given in the sections below.

### DIA Mass Spectrometry: Peptidomics and Label-Free Proteomics

Peptidomic and proteomic analyses were performed on a nanoACQUITY UPLC system coupled to a SYNAPT G2 mass spectrometer (Waters) operating in DIA mode, using MS^E^ for peptidomics and MS^E^/HDMS^E^ for label-free proteomics. Chromatographic separations employed 0.1% formic acid (FA) in water (mobile phase A) and 0.1% FA in ACN (mobile phase B).^23,29^ Peptides (2 μg for peptidomics and 7 μg for proteomics) were eluted with a 5–35% B gradient over 12 or 30 min for peptidomic fractions and 60 min for proteomic samples.

Mass spectra were acquired in resolution mode.^20,23^ Fragmentation was achieved by alternating low-energy (4 eV) and elevated-energy scans using collision-energy ramps of 23–58 eV for MSE and 15–65 eV for HDMSE acquisitions. All DIA LC-MS/MS experiments were conducted at the Laboratory of Mass Spectrometry Applied to Biomolecules (LEMBio/UNIFESP).

### DIA Quantitative Analysis

Label-free quantification was performed in Progenesis QI for Proteomics (Nonlinear Dynamics, UK) as previously described.^20,23,30^ Peak lists were searched in PEAKS Studio v7.5 (Bioinformatics Solutions Inc.) against a species-specific *C. d. terrificus* protein database comprising 237 nonredundant entries (January 25th, 2022). Peptide identification employed precursor and fragment mass tolerances of 10 ppm and 0.025 Da, respectively, with carbamidomethylation as a fixed modification, methionine oxidation and N-terminal pyroglutamate formation as variable modifications, up to two missed cleavages, and a peptide-level FDR of 1%. Quantitative data were imported back into Progenesis, and peptide abundances were determined from integrated ion intensities following manual inspection of quantitative features.^23^

### DIA Native Peptidomic Analysis

Peptidomic analysis was performed as described above, except that enzymatic specificity and fixed modifications were not employed. Peptides identified in database the search were manually reviewed to verify ion charge, monoisotopic peak assignment, and to validate that the mass difference in ppm was lower than 20.^23^ Peptides presenting a minimum of 5 fragments matching *b-* or *y-*ion series^31^ were considered for further analysis. Theoretical mass of ions was calculated in ProteinProspector v6.4.5. Peptides with ALC scores ≥ 80% that were not identified by automated database searches were subject to manual *de novo* sequencing,^26,32^ adopting the same parameters.

### DDA Mass Spectrometry: TMT Proteomics

Samples were analyzed in duplicate by nanoLC-MS/MS (Thermo Scientific) using an EASY-nLC 1200 coupled to a Q Exactive HF-X Orbitrap. Peptides (2 μg) were loaded onto an Acclaim PepMap C18 trap column (75 μm × 2 cm, 3 μm, 100 Å) at 2 μL min^–1^ in 0.1% FA and separated on a 200 cm μPAC RP18 column (PharmaFluidics) at 45 °C. Mobile phases consisted of 0.1% FA in water (A) and 0.1% FA in 80% acetonitrile (B). Peptides were eluted at 400 nL min^–1^ with a gradient from 1% to 97% B over 142 min, followed by a 10 min hold at 97% B.^23^ MS acquisition was conducted in positive-ion mode.^23^ Survey scans were acquired from *m*/*z* 350–1500 at 60,000 resolution (AGC 3 × 10^6^; maximum injection time 50 ms), and the 20 most abundant precursors were selected for HCD fragmentation (0.7 *m*/*z* isolation window; normalized collision energy 32). MS/MS spectra were recorded at 45,000 resolution (AGC 1 × 10^5^; maximum injection time 96 ms). Dynamic exclusion was set to 30 s, and ions with undefined charge states, *z* = 1, or *z* > 5 were excluded. All DDA experiments were performed at the Proteomics/RJ Technological Platform (RPT02), Fiocruz.

### TMT Quantitative Proteomics

Peptide identification and reporter ion quantification were carried out in Proteome Discoverer (v3.0, Thermo Scientific). For sequence identification, MS/MS spectra were searched with SEQUEST HT against the *C. d. terrificus* database, previously described, complemented with 251 common contaminant sequences. Search parameters allowed up to two missed tryptic cleavages, considered peptides between 6 and 144 residues in length, and employed precursor and fragment tolerances of 10 ppm and 0.025 Da, respectively. Carbamidomethylation and TMT labeling were treated as fixed modifications, whereas methionine oxidation and N-terminal pyroglutamate formation from glutamine and glutamic acid were considered variable modifications. Identification confidence was controlled by limiting accepted peptide-spectrum matches to a false discovery rate of 1%. TMT reporter signals were extracted using a 10-ppm mass window, and quantitative comparisons were restricted to razor and protein-unique peptides. Abundance values were first adjusted according to total peptide signal and subsequently normalized against the pooled internal standard assigned to channel 126.^33^

### Data Imputation and Processing

Quantitative data generated by DIA and DDA workflows were analyzed in R (v4.2.0). Entries exhibiting ≤ 50% missing values were retained and imputed by random forest (*rfImpute*, *randomForest* v4.7–1.1) using 1,000 trees and 10 iterative cycles.^34,35^ Batch-related variation in DDA experiments was adjusted with the ComBat method from the *sva* package.^36^

### Variant Analysis

All mass spectral data (DIA and DDA) were analyzed for variant detection using the SPIDER module of PEAKS Studio 7.5, using the same parameters as the database searches for each data type. To confidently assign amino acid residue substitutions, a critical manual validation of MS data^37^ was performed taking into account the following criteria:^23^ (i) no missed cleavage allowed, using semispecific digestion mode; (ii) the assigned sequence presented only expected modifications; (iii) the substituted residue needed to be supported by complete and consistent *b*- and *y*-ion series coverage; (iv) mass spectral peaks above the noise level; (v) discernible monoisotopic peak with no distribution overlapping; (vi) more than one high quality spectra supporting the mutation; (vii) the corresponding unsubstituted (native) peptide had to be identifiable in a standard database search to confirm peptide uniqueness.^23^ All validated variations were consolidated into a custom variant database, which was subsequently used in a second-round database search using the respective processing workflows for quantitative analysis.

### Statistical Analysis

Differences in abundance between groups were evaluated using two-sided Student’s *t*-tests or Welch’s *t*-tests, depending on variance homogeneity.^39^ Differentially abundant features were defined by *p* < 0.05 and |log_2_FC| > 0.3.

### Peptide Alignments

The primary structure of selected peptides was analyzed using BLAST (blast.ncbi.nlm.nih.gov/Blast.cgi) to search nonredundant protein sequences of *Crotalus* (taxid: 8728) and Serpentes (taxid: 8570).^23^ Sequence alignment was performed in Clustal Omega (ebi.ac.uk/Tools/msa/clustalo/) and visualized in Jalview.^40^

### Protein Folding and Visualization

The three-dimensional structures of proteins were predicted by AlphaFold 2.0 ^41^ using Docker (github.com/deepmind/alphafold). For multiple-sequence alignment template searches on PDB70, Big Fantastic Database, and UniRef30, we used HHSearch/HHBlits. For UniRef90 and clustered MGnify, we used Jackhmmer. The available templates released on January first, 2023, were used in this study. Constrained relaxation of structures was performed using OpenMM with the Amber99sb force field.^41^ Models with the highest per-residue confidence metrics were selected and compared with experimentally determined structures of species-specific proteoforms or, when unavailable, to the most closely related homologous protein sequence accessible in the Protein Databank (PDB).^23^ Mutations occurring in the signal peptide or on unresolved crystal residues were not subjected to structural prediction. All-atom alignment and structure rendering were performed in PyMOL Molecular Graphics System, v.2.4.1 (Schrödinger, LLC). The effect of missense mutations on protein stability was predicted by ΔΔ*G* Stability upon single and multiple point mutations using the DDMut^42^ Web server (biosig.lab.uq.edu.au/ddmut).

## Results and Discussion

### Compositional Analysis (SDS-PAGE)

The overall electrophoretic venom profile of the specimens used in this study presented five main protein bands with molecular masses of approximately 56 kDa, 29 kDa, 14 kDa, and 10 kDa (Figure 2), indicating that the venom composition remains relatively consistent across individuals in SDS-PAGE analysis. Despite differences in intensity, the 14 kDa and 30 kDa bands were detected in all venom samples, whereas notable variability was observed in the 56 kDa and 10 kDa bands. In addition, a comparable band composition can be observed in both reducing conditions (), although subtle exclusive bands have been reported under these conditions in *Crotalus durissus* venom.^13^ The profiles were similar to those found when investigating 315 individual samples of *C. d. terrificus* (Cdt) specimens.^15^

**Figure 2:**
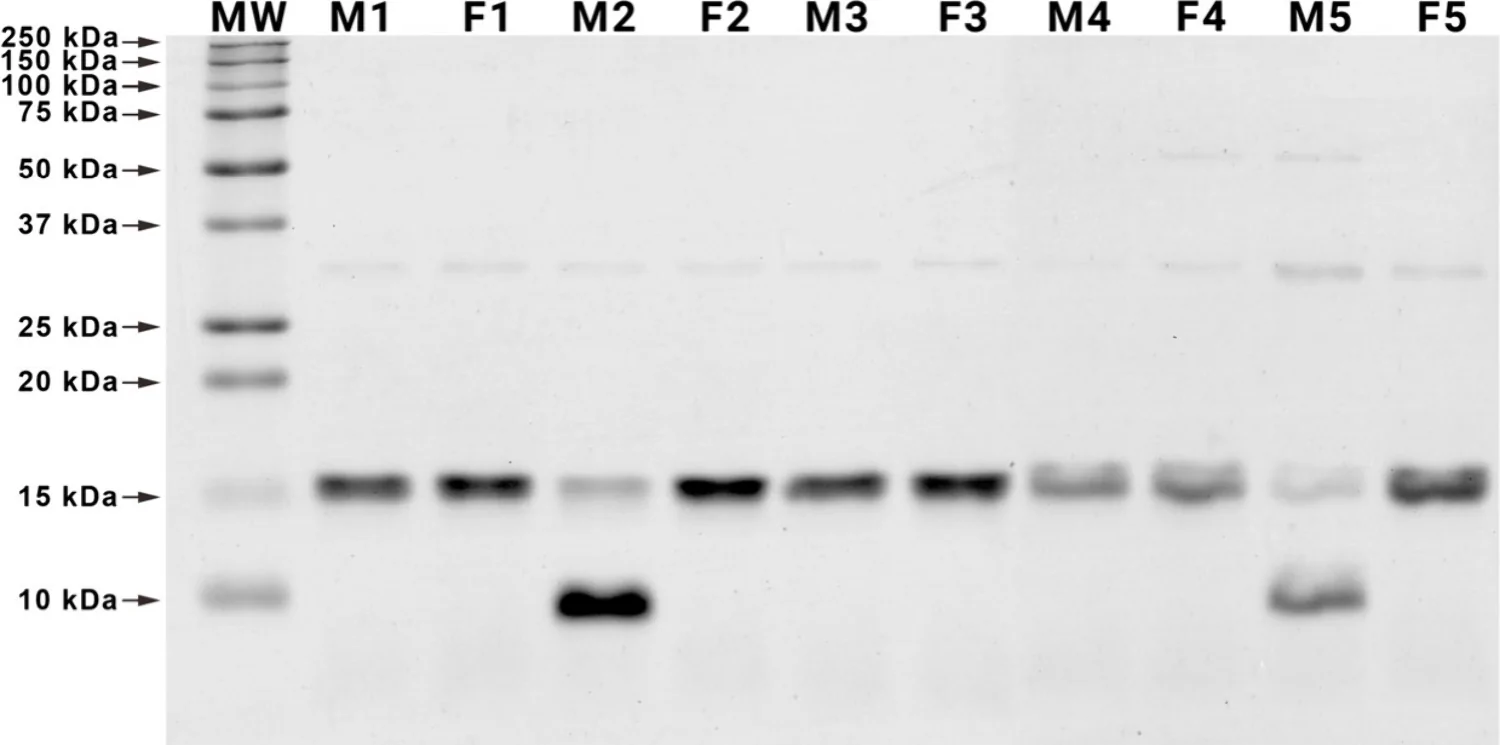
Electrophoretic profile of individual venom samples (10 μg) subjected to SDS-PAGE 15% under reducing conditions. MW: molecular weight reference marker (Precision Plus Protein – BioRad), M: male, F: female.

As *C. durissus ssp.* venoms have been deeply characterized, and protein bands can be assigned based on their molecular masses.^11,23^ The main protein bands found in Cdt venom samples (∼14 kDa and 29 kDa) correspond to CB and thrombin-like enzyme gyroxin, respectively. Two specific protein bands showed variation in their presence among specimens, in ∼ 56 and 10 kDa, generally assigned to L-amino acid oxidases (LAAO), and crotamine, respectively (Figure 2).^23^ As the run was stopped early to retain the low-mass crotamine within the gel, the low-molecular-mass region was compressed and the corresponding apparent masses are approximate. Therefore, crotamine (∼5 kDa) migrates close to the 10 kDa marker. LAAO protein bands were observed in two individuals, one female (F4) and one male (M5). Crotamine, on the other hand, was detected in only two male individuals (M2 and M5).

### Relative Quantitative Peptidomics

The native peptides of Cdt venom were submitted to MS^E^ DIA analysis. Automated *de novo* analysis of 6,931 MS/MS spectra resulted in 325 peptide-spectrum matches. After manual revision of peptide assignments, 76 peptides were validated and retained from the database search. Crotoxin represented the dominant originating protein, contributing to 48 (63%) of all identified peptides (), with 33 originating from the basic subunit (CB - P0CG56.1: P24027.1: P62022.1) and 15 from the acidic subunit (CA - P08878.1). Crotoxin is the major component of Cdt venom, consisting of a noncovalent heterodimer formed by CB (neurotoxic phospholipase A_2_) CA (nonenzymatic crotapotin) subunits.^23,43^ Following crotoxin, snake venom serine proteinases (SVSP) and snake venom metalloproteinases (SVMP) accounted for 12% and 8% of identified peptides, respectively.

Interestingly, previously described small pyroglutamyl/prolyl peptides^44^ and BPPs^45,46^ were not detected in our peptidomic analysis.^23^ The absence of these peptides can be attributed to the almost negligible abundance of CNP/BPP in Cdt venom.^47,48^ However, other pyroglutamyl peptides (pGlu or Z) originating from different precursors have been identified in the venom peptidome of Cdt. Nine pGlu peptides derived from CA, CB, the hyaluronidase CdtHya1, and the SVMP MAD-3a were found (). We highlight the identification of the peptide ZNWESNEPI from MAD-3a, as it originated from an SVMP, contains the ZNW sequence, as found in other BPPs, and exhibits similarities to the peptide Bc-7a (ZRYDPFR), identified in the venom of *Bothrops cotiara*,^49^ and to LM-10a (ZRFSQKYIEL), identified in the venom of *Lachesis muta*.^29^ The cleavage site C-terminal to ZNWESNEPI has lysine at the P1′ position (), analogous to the C-terminus of Bc-7a (-PFR), where arginine occupies the P1 position.^49^ These structural parallels suggest that other SVMP-derived peptides from Viperidae venoms may also exhibit similar bioactivity, given that Bc-7a has been shown to function as an angiotensin-converting enzyme inhibitor.^49^

Remarkably, several peptides detected in Cdt venom originate from the processing of specific regions within the precursor. For instance, peptides spanning residues 17–29 of CA (signal peptide) and 17–41 of CB (the N-terminal region of the mature toxin) were identified. The database search also led to the identification of complete β (residues 84–118: ZEDG­EIVCG­EDDPC­GTQIC­ECDKA­AAICF­RNSMDT) and γ (125–138: ZFSPE­NCQGE­SQPC) chains of CA (), as reducing agents disrupted the three disulfide-linked polypeptide chains (illustrated in ).

Furthermore, 9 novel peptides were manually *de novo* sequenced. The homology search of these novel peptides did not yield significant alignments with known Crotalinae venom toxins among the top-scoring hits (), except for FTLP­[I/L]­FVNPVN, which aligned with the venom phosphodiesterase of *C. adamanteus* (Accession: J3SEZ3.2) with 100% sequence identity and an E-value of 0.001.^23^

Quantitative peptidomic analysis revealed five peptides with different abundances between sexes. Two peptides derived from the CA β-chain (CYCGAG­GQGWPQ and CGAG­GQGWPQ) identified by database search () showed increased abundance in females (FC of 1.70–2.23), whereas three novel peptides (HLLHLLQ, SSYGHLLQ, and QHLLHLLQ) identified by *de novo* analysis () were more abundant in males (FC 1.98–3.25), as depicted in Figure 3.

**Figure 3:**
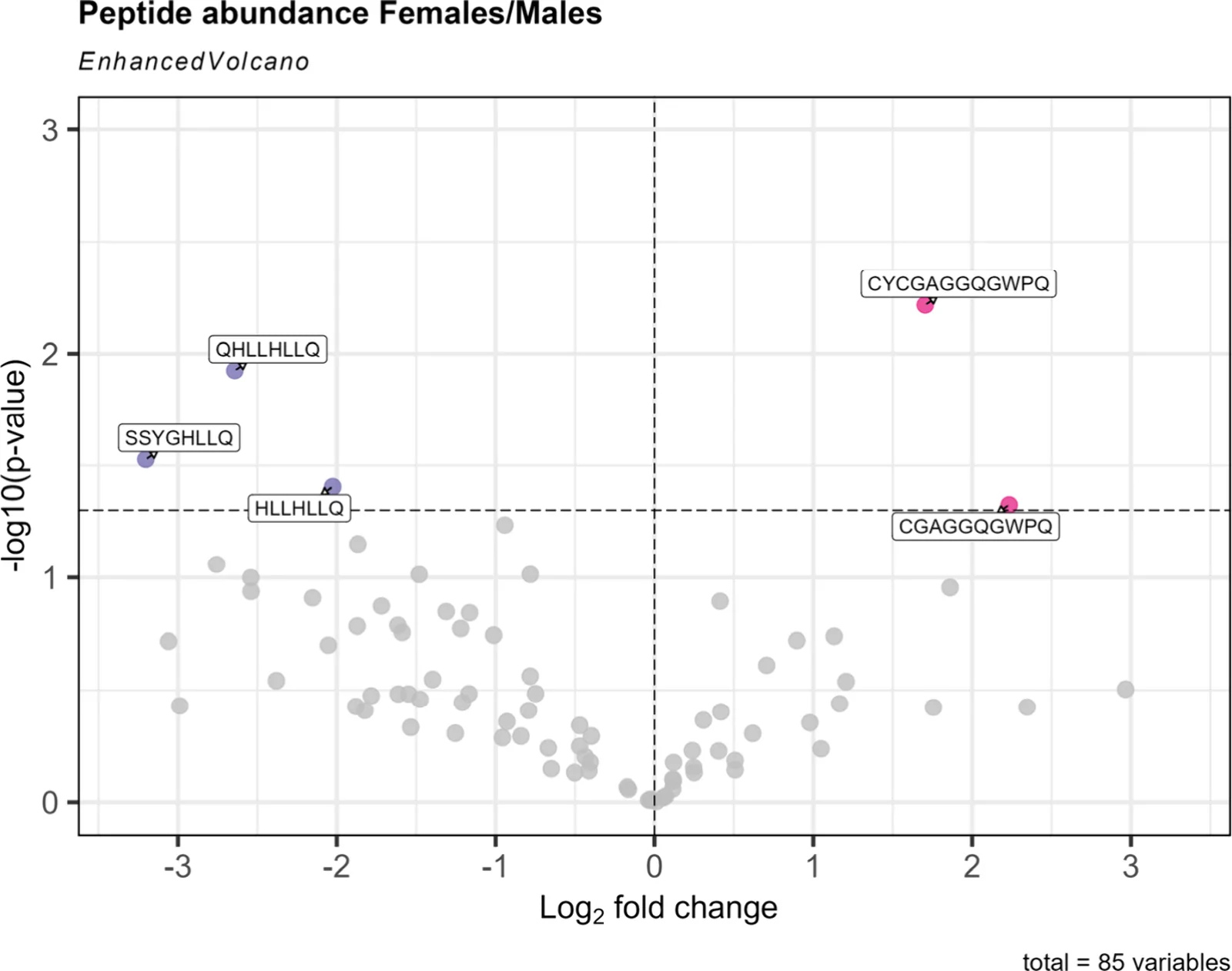
Volcano plot of peptide ions identified in C. d. terrificus venom. Fold changes were calculated as the average abundance ratio of females to males and expressed on a log2 basis. Peptides above the significance threshold are depicted in the upper left panel (blue, higher abundance in males) and upper right panel (pink, higher abundance in females). The volcano plot was built in R using the “EnhancedVolcano” package.

Upon closer examination of the differentially abundant novel peptide sequences, a notable degree of sequence superposition was observed, particularly in the putative motif HLLQHLLHLLQ (Figure 3 and ). This recurring pattern gives rise to a hypothetical sequential tandem array that resembles the N-terminal of Viperidae PLA_2_. This finding suggests that these novel peptides may originate from shared uncharacterized PLA_2_ precursors.^23^

### Proteolytic Processing of Cdt Peptides

Proteolytic analysis was performed by mapping N- and C- terminal flanking residues up to positions P4–P4’ on full-length protein sequences to reconstruct the cleavage sites of identified peptides^50^ in native peptidomic analysis.^23^ Based on amino acid frequencies of identified peptides ( and Figure 4), the analysis reveals a high prevalence of N-terminal glycine (29%), valine (28%), glutamic acid (28%) and glycine (29%) at positions P4–P1. This reflects reproducible processing of the N-terminal segments of CA (GVEGSLVE, P4–P4’ 13–20) and CB (GVEGH/SLLQ, P4–P4’ 13–20). The strong enrichment of leucine at P3–P1 further supports recurrent cleavage at the leucine pair (H/SLLQ) within the CB N-terminus. On the C-terminal side of the detected peptides, the high frequency of phenylalanine P2’ (26%), again indicates preferential cleavage of CB (17–41). Collectively, these patterns demonstrate that peptides in Cdt venom may define proteolytic signatures, reproducibly generated at specific amino acid positions within mature toxins.

**Figure 4:**
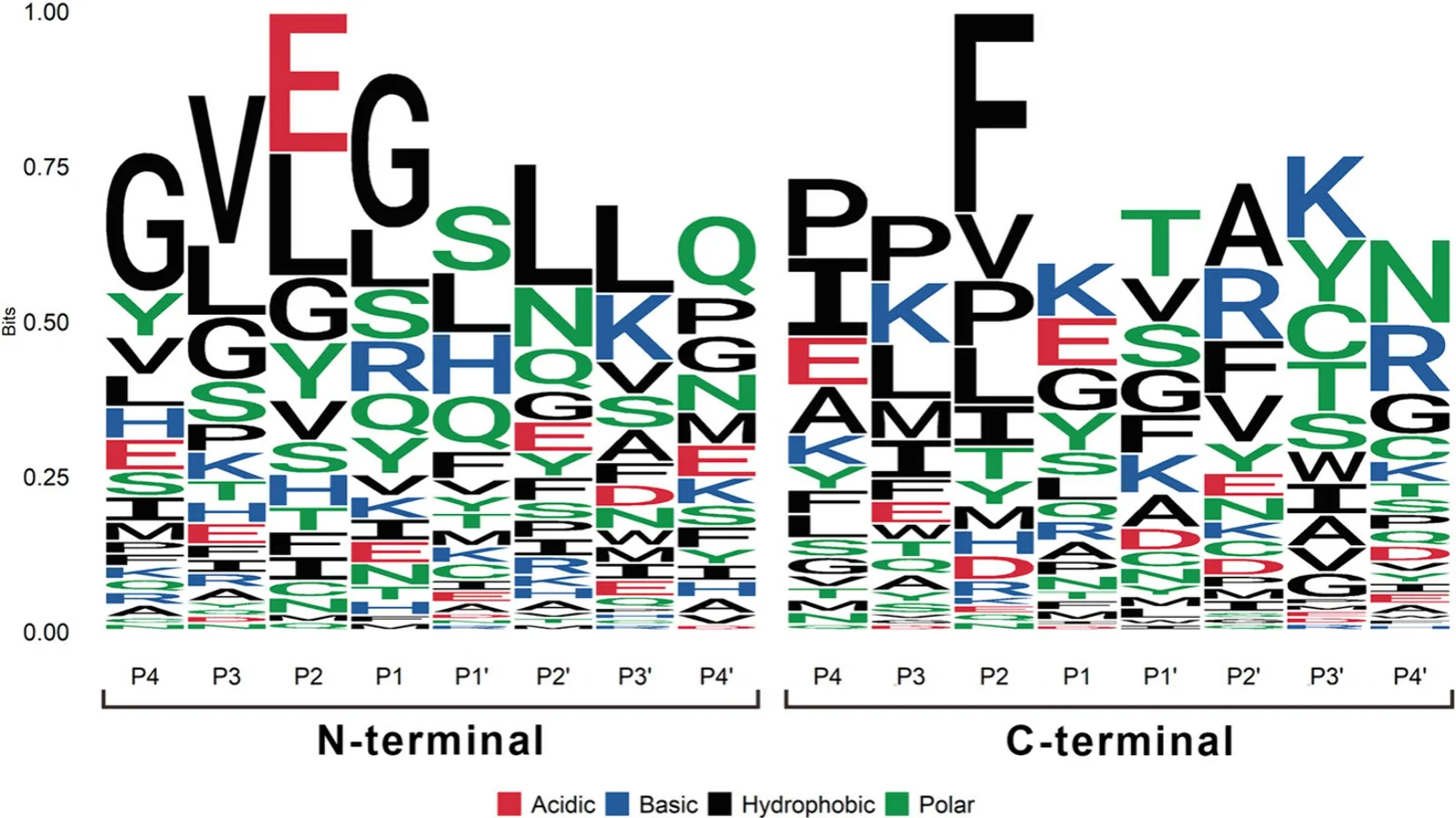
Amino acid frequencies at positions P4–P4’ of N and C-terminals of identified peptides in the venom peptidome of C. d. terrificus. The ice logo plot was built in R using the “SeqLogo” package.

### Quantitative Proteomics

Quantitative proteomics of Cdt venom was carried out using TMT-DDA (Tandem Mass Tag-Data-Dependent Acquisition).^23^ Through proteomic analysis of Cdt venom, 33 proteins were identified, belonging to 16 protein groups (). The venom exhibited a typical predominance of crotoxin, crotamine, SVSP (gyroxin), CTL (including crotocetin and convulxin), LAAO (bordonein-L), SVMP (including P–III MAD-3a, crotastatin, and P–II MDC-2) and VEGF (VEGF 1, 3 and 4), as illustrated in Figure 5A. Other minor venom components were also identified, each constituting less than 1% of total relative abundances, including hyaluronidase, phosphodiesterase (PDE), cysteine-rich secretory protein (CRISP), phospholipase B (PLB), aminopeptidase, bradykinin potentiating peptide (BPP) precursor, and phospholipase inhibitor.^23^

**Figure 5:**
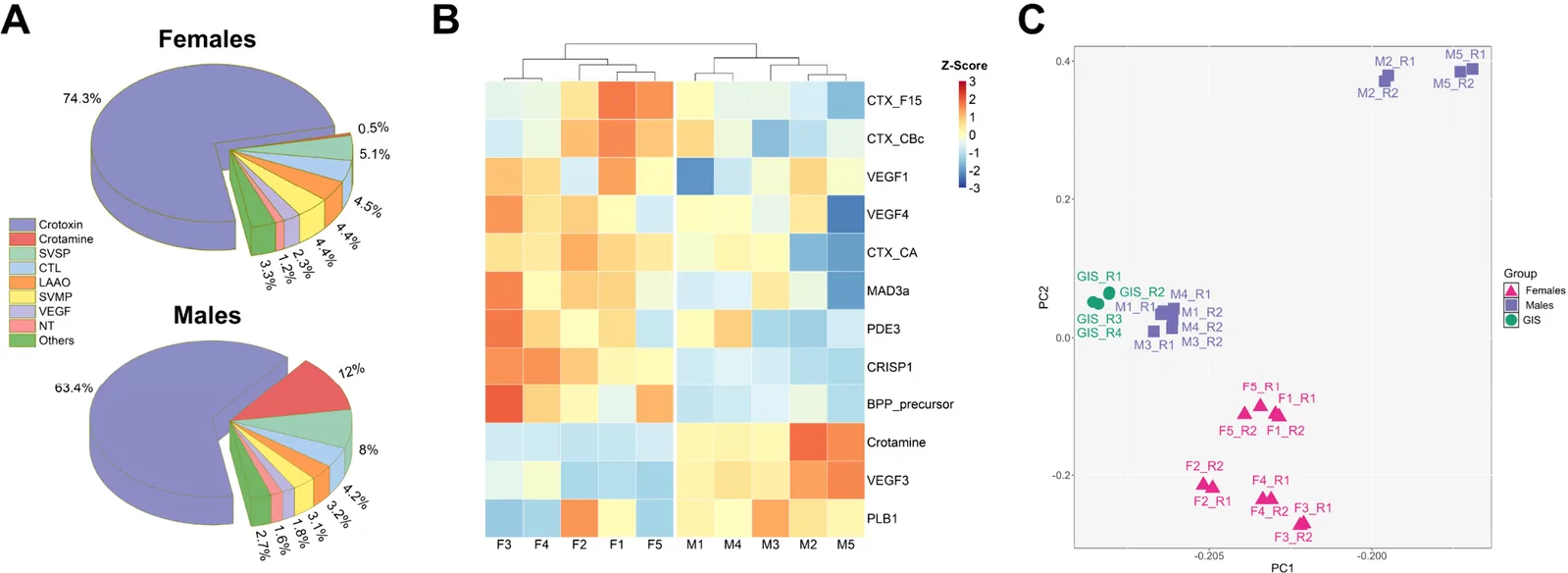
Quantitative proteomics. (A) Overview of relative protein abundances in *C. d. terrificus* venom according to protein families, comparing females (upper pie chart) and males (lower pie chart). (B) Heatmap depicting differentially abundant proteins between males and females. (C) Principal component analysis (PCA) from DDA-TMT proteomics. Controls (pooled venoms) are shown as green circles, female venoms as pink triangles, and male venoms as blue squares. Samples were analyzed in biological quintuplicates and technical duplicate runs. Pie charts were built in OriginLab, heatmap in R using “pheatmap” package, and PCA using “ggplot2” package.

Among identified proteins, 12 exhibit significant sex-related differences in abundances ( and Figure 5B), representing approximately one-third of the total venom proteome. Crotoxin was the most abundant component, with relative abundances ranging from 63.4% in males to 74.3% in females. Notably, females exhibit higher levels of both CA and CB subunits (FC of 0.72 and 0.97, respectively). These findings are consistent with the increased abundance of CA-derived peptides in females observed in the peptidomic analysis. Other proteins enriched in female venom included VEGF1, VEGF4, MAD-3a (SVMP), PDE3, CRISP1, and BPP precursor, with FC ranging from 0.31 to 3.83 ( and Figure 5B). In contrast, males displayed markedly higher levels of crotamine, with an average relative abundance of 12%, compared to only 0.5% (FC of 4.35) in females. Additionally, male-enriched proteins included VEGF3 and PBL1, with FC values of 0.62 and 0.51, respectively.

The relatively permissive fold-change cutoff (|log_2_ FC| > 0.3) was adopted to account for TMT ratio compression, a well-documented attenuation of reporter-ion ratios caused by coisolation of interfering precursors in MS2-based isobaric quantification, which underestimates true fold changes.^51,52^ This threshold preserved sensitivity to coordinated shifts across toxin classes, exemplified by the consistent female-directed changes among crotoxin proteoforms (), which reflect a broader compositional reorganization rather than isolated large-effect differences.

The distinct proteomic profiles of male and female venoms were further supported by principal component analysis (Figure 5C).^23^ The clustering pattern clearly separated controls (pool), males, and females. A secondary cluster composed of two male individuals paralleled the distinct presence of ∼ 10 kDa protein bands of M2 and M5 (Figure 2), corresponding to the high abundance of crotamine in these individuals (35% and 20% of total protein abundance, respectively, ). Correspondingly, the intraspecific variation in the 56 kDa protein bands of F4 and M5 correlated with increased LAAO abundance on these individuals (10–12%), compared to the average abundance in other specimens (2%).

### Variant Analysis

Variant and homology search resulted in 242 (DIA proteomic and peptidomic analyses) and 905 (DDA-TMT proteomic analysis) reported peptide variants. After manual inspection, a total of 16 peptides describing 12 variants were validated (, which showcase variant spectra, Table 1 and ). Detected amino acid variants primarily occurred in major Cdt venom toxins, including crotoxin CA and CB subunits, and gyroxin. Interestingly variant detection in CA was restricted to DIA, whereas variants in CB were detected only in DDA, highlighting the complementarity between acquisition methods and instrument platforms. The only convergent variant detection among acquisitions was the S136N substitution in gyroxin (Table 1), identified in both DIA peptidomic and DDA proteomic analyses.

**Table 1:**
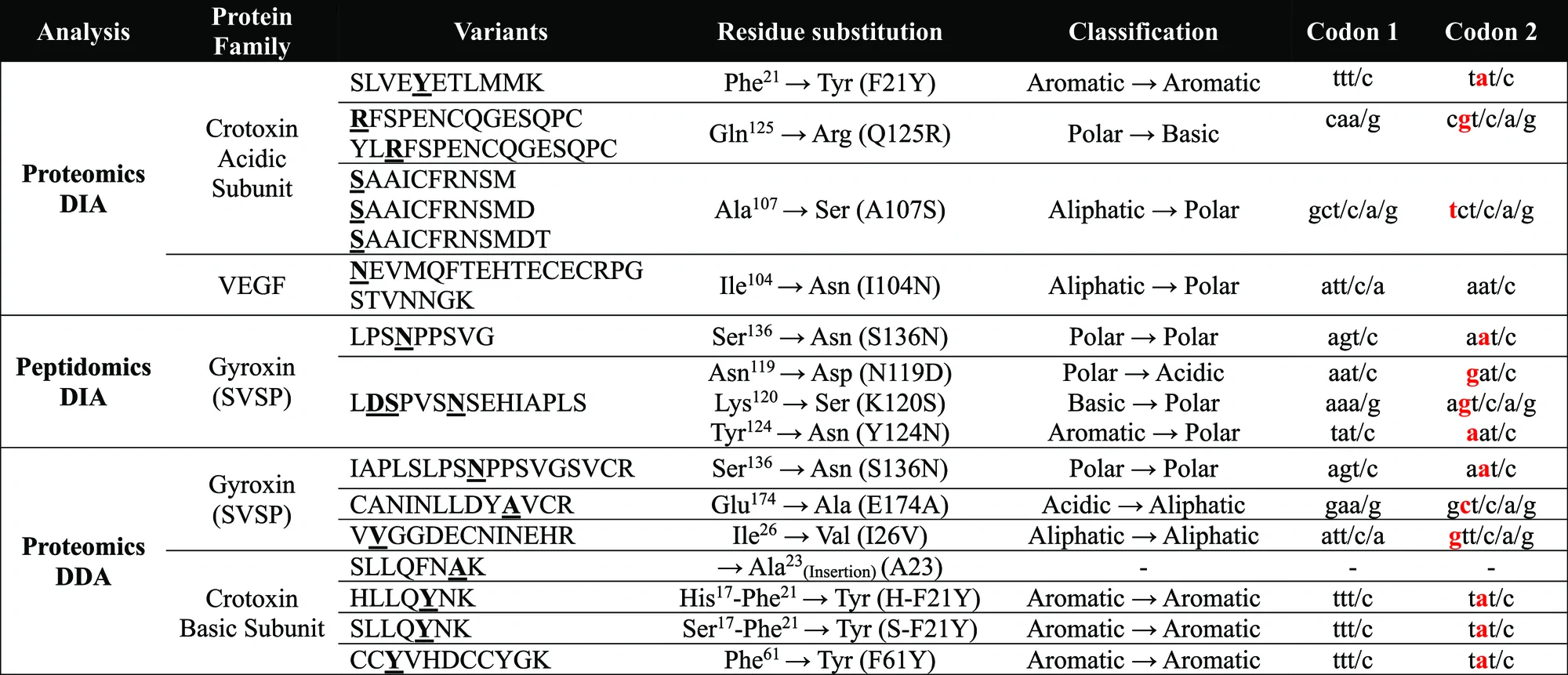
Sequencing Validation of Amino Acid Variants by Mass Spectrometry and Translation Code of Amino Acid Variants^a^

a Potential single nucleotide polymorphisms are highlighted in red.

The observed variants primarily consisted of single-residue substitutions. This observation aligns with well-established knowledge that the most prevalent type of protein variant in biological systems corresponds to single-amino acid variants.^53^ Furthermore, all residue substitutions except for I104N (VEGF4) can be attributed to single-nucleotide polymorphisms (SNP), as indicated in Table 1. These associations are drawn from the transcriptome shotgun assembly sequences of Cdt^48^ and the standard translation code provided by the NCBI taxonomy browser (NCBI txid 8732).^23^ Remarkably, residue substitutions were not limited to neutral replacements and, in several cases, resulted in changes in the polarity of the encoded amino acid side chains, as illustrated in Table 1. In contrast to point mutations, the insertion of alanine^23^ (CB) may result from a nucleotide insertion event, another key mechanism in venom diversification and adaptive variation.^54^

Peptide variants of the crotoxin acidic subunit were aligned to the CA precursor: variants F21Y is on the signal peptide, A107S on the β-chain, and Q125R on the γ-chain, as represented in Figure 6A. Insertion of A23_,_ F21Y, and F61Y are depicted in Figure 6B. Interestingly, the residue substitutions did not occur in crotoxin key contact residues,^43,55^ suggesting that the observed mutations may not significantly impact the overall stability and toxicity of this neurotoxin.^23^ The homologous search for Q125R substitution in CA resulted in a significant alignment with the crotoxin acidic chain of Mojave toxin of *C. s. scutulatus* (Acession: P18998.2), with 100% sequence identity (*E*-value: 2 × 10^–09^). Supporting the single residue substitutions found in crotoxin, Mojave toxin evolution toward neurotoxic activity occurred through a gradual cascading of diversification unlocked by a single SNP.^23,56^ Additionally, a similar substitution of arginine replacing glutamine (Q136R) has also been previously reported in Cdt CA^57^ at the proteoform level.

**Figure 6:**
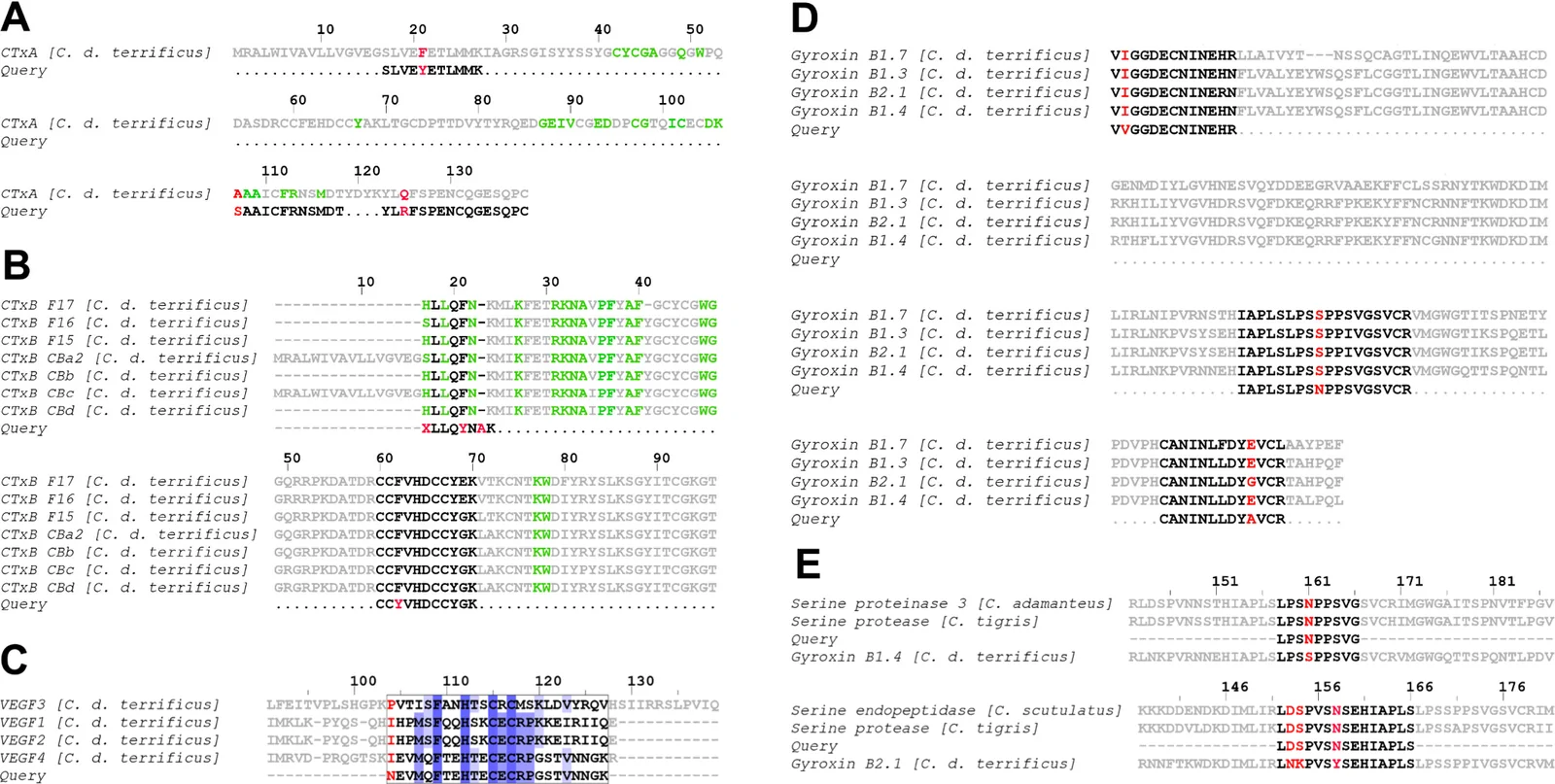
Variant analysis. Variant peptides of crotoxin acidic (A) and basic subunit (B). Variants are represented in black, substituted residues in red, contact residues in green. “X” denotes histidine or serine N-terminal residues of CB proteoforms. Asparagine residue substitution in CdtVEGF-4 is shown in (C). Residue conservation within the alignment region is indicated in blue as percentage identity. Variants identified in gyroxin by proteomic DDA analysis (D) or peptidomic DIA (E) are also presented. Residues 32–93 in panel (D) are omitted for clarity.

The Cdt venom VEGFs exhibit a remarkable degree of complexity in terms of their abundance, precursor processing, and structural domains.^48^ Nevertheless, the specific substitution I104N (Figure 6C) occurred in a highly variable region located within the platelet-derived growth factor domain.^48^ Although this was the only residue substitution not attributable to a SNP, the same variation between isoleucine and asparagine can be observed when comparing aligned positions 125 of CdtVEGF-1/2 and VEGF-4 (Figure 6C), which also does not constitute a SNP,^48^ indicating that it might be a common residue variation among Cdt-VEGFs.^23^

Alignments of peptides identified in Cdt gyroxin show variants involving valine and isoleucine (VIGGDECNINEHR and IVGGDECNINEHR), representing subtle yet commonly overlooked variation in many snake SVSPs subjected to accelerated evolution.^23,58^ Nonetheless, this substitution (I26V) exhibited significant alignment with Viperidae SVSPs (Figure 6D), including P0DMH5.1 from *Bothrops cotiara* (100% identity and E-value of 3 × 10^–08^). Furthermore, Figure 6E demonstrates significant alignments between variants found in gyroxin, identified through DIA peptidomic analysis, and homologous SVSPs from American pit vipers. These alignments exhibit sequence identity of 100% and E-values ranging from 6 × 10^–09^ to 8 × 10^–03^, suggesting that the observed substitutions in SVSP are naturally found within *Crotalus* species. Nevertheless, it is crucial to note that N199D represents a common nonenzymatic chemical modification of proteins and peptides both *in vivo* and *in vitro*.^59^ Therefore, the possibility of spontaneous deamidation of asparagine cannot be disregarded.^23^

Previous transcriptomic analysis of *C. d. terrificus* venom glands revealed substantial variation in paralogous transcript abundances.^48^ However, Cdt venom proteomic analysis has focused primarily on grouped protein families, with limited attention to properly addressing proteoforms.^13,48,60–63^ To accurately assign proteoforms in mass spectrometry analysis, quantifying unique peptides has proven to be the most reliable approach.^64,65^ Quantitative analysis did not indicate significantly abundant variant peptides in any sex. Consequently, peptide variant abundances were compared with their native counterparts (equivalent peptides of known sequences assigned in database search), across all individuals (males and females). As a result, eight differently abundant peptide variants were observed (Figure 7), referring to substitutions on CA (F21Y, Q125R and A107S; FC 1.99–4.46), CB (A23, H-F21Y and F61Y; FC 0.75–4.49), gyroxin (I26V; FC 1.65) and VEGF (I104N; FC −2.17).

**Figure 7:**
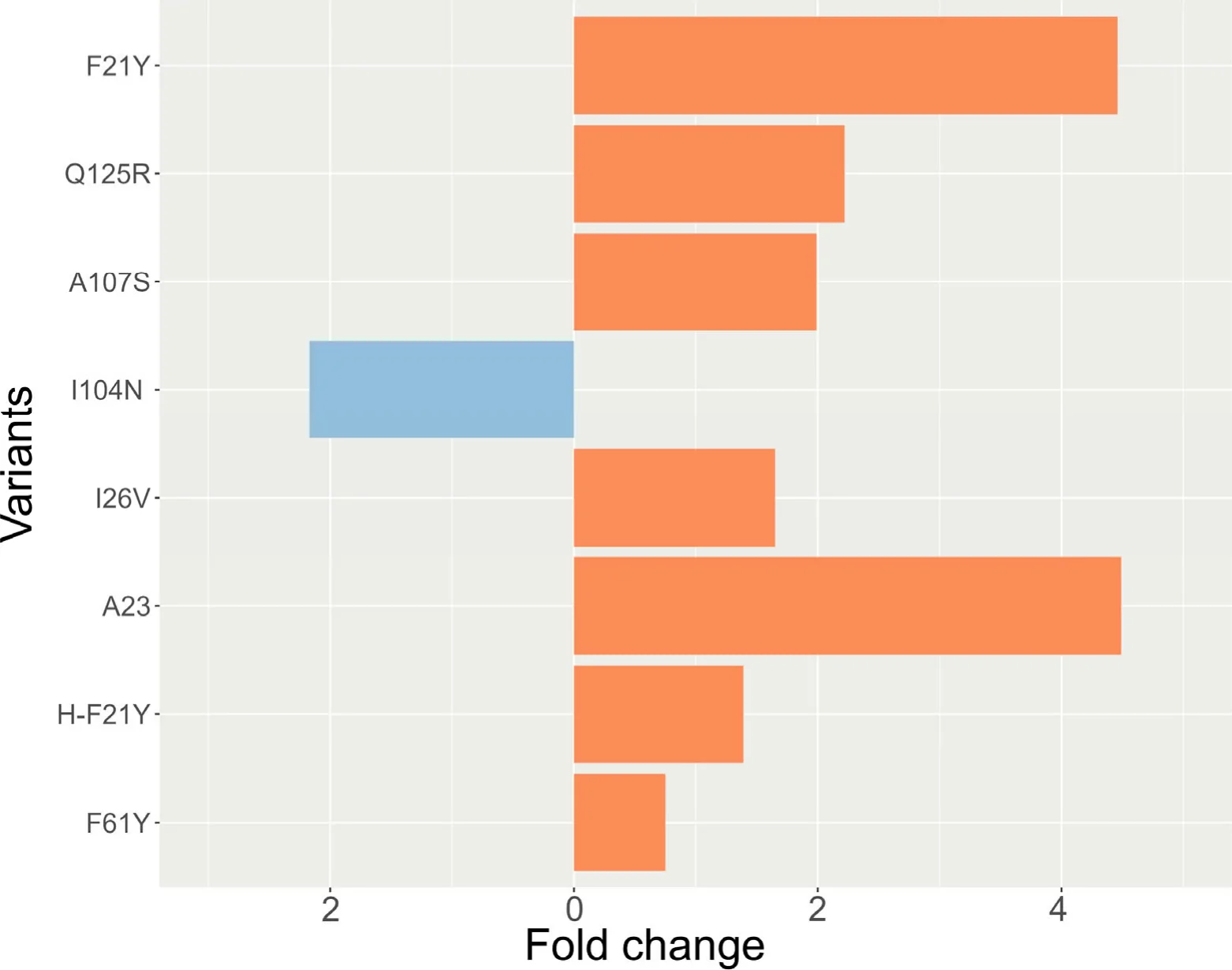
Comparison between native and variant peptides. Fold changes were calculated as the average peptide ion abundances of native/variant peptides on log_2_ basis. Significantly higher abundances of native peptides and variant peptides are colored orange and blue respectively. The bar graph was built in R using “ggplot2” package.

### Impact of Mutations on Three-Dimensional Structures and Protein Stability

To investigate the impact of mutations on the three-dimensional structure of toxins, the protein folding computational tool, AlphaFold 2,^66^ was employed to predict the structures of variant toxins.^23^ The predicted variant structures of the CA β-chain α-helices (Figure 8A) and CB subunit (Figure 8B) showed comparable amino acid residue orientations between predicted structures and the respective native crystal. The insertion of A23 in CB led to a widening of the helix groove by approximately 2.4 to 3 Å (Figure 8C), while still preserving the N-terminal helix A of CB,^43^ as α-helices can accommodate three to four residues per turn.^67^ Similarly, the predicted structures of variant VEGF (Figure 8D) and gyroxin (Figure 8E-G) superpositioned onto their respective homologous sequences revealed a remarkable alignment to crystal structures.

**Figure 8:**
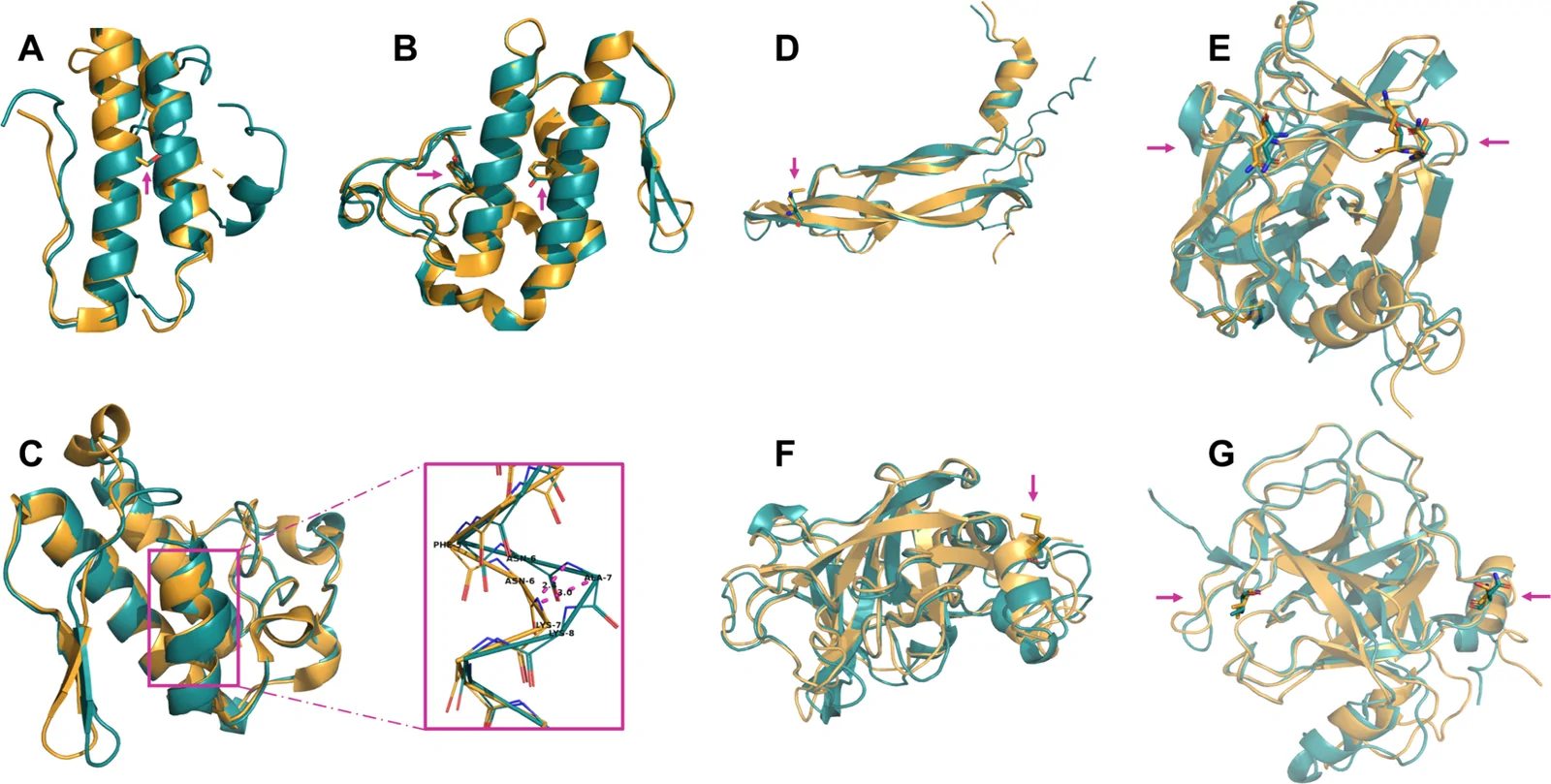
Prediction of amino acid variation impact on toxins three-dimensional structure. The A107S substitution in the crotoxin β-chain acidic subunit is shown in (A). The S-F21Y (left arrow) and F61Y (right arrow) substitutions in the crotoxin basic subunit CBc are shown in (B). Alanine insertion A23 in the crotoxin basic subunit CBa2 is shown in (C). The I104N substitution in VEGF-4 is shown in (D). In gyroxin B2.1, a triple substitution—Y124N, K120S, and N119D (from left to right)—is shown in (E) along with the Q174A substitution in (F). The I26V (left arrow) and S136N (right arrow) substitutions in gyroxin B1.7 are also depicted in (G). Predicted structures were superimposed onto 3R0L and 2QOG from *C. d. terrificus* and homologous 5XRF and 1WQ8 from *D. acutus* and *V.* a. aspis., respectively. Crystal sequences are depicted in orange, variant sequences in cyan, shown overlaid as cartoon, purple arrows indicate residues substitutions, interatomic distances are depicted by purple dashed lines in angstroms.

The per-residue alignment of predicted variant structures onto their corresponding crystal structure resulted in relatively small root-mean-square deviation values of superimposed residues, reaching as low as 0.05 Å (as detailed in Table 2). In addition, mutated residue domains presented high-confidence Predicted Local Distance Difference Test (pLDDT > 80%) scores. This outcome underscores a low degree of localized structural deformation arising from residue substitutions and high accuracy of the prediction,^41^ as AlphaFold 2 has proven to be a powerful tool for predicting local structure changes due to single mutations in proteins, when the average pLDDT is above 70 and substitutions are located in defined secondary structures.^23,68^

**Table 2:** Structural Analysis of Amino Acid Variants

**Residue substitution**	**Aligned structure (PDB ID)**	**Average Local RMSD (Å)**	**Individual ΔΔ*G* Stability** ^a^	**Global ΔΔ*G* Stability** ^a^
A107S	3r0l	0.05	+0.05	NA
H-F21Y	2qog	1.45	+0.02	+0.6–0.62
F61Y	2qog	1.46	–0.71	
S-F21Y	2qog	1.45	–0.08	
S136N	5xrf	0.71	–0.16	+0.37
I26V	5xrf	1.95	+0.42	
E174A	5xrf	0.86	–0.04	+1.36
N119D:K120S:Y124N	5xrf	0.88	–0.11:+0.1:0	
I104N	1wq8	0.11	–0.26	NA

a Stabilizing mutations (ΔΔ*G* ≥ 0 kcal/mol, “+”), destabilizing mutations (ΔΔ*G* ≤ 0 kcal/mol, “–”).

In contrast, individual assessment of missense mutations (Table 2) elucidates a spectrum of mutational effects, ranging from stabilizing (ΔΔ*G* Stability ≥ 0 kcal/mol) to destabilizing (ΔΔ*G* Stability ≤ 0 kcal/mol) alterations on protein stability.^42^ However, when extending the investigation to encompass multiple mutations, a global stability perspective emerges, revealing an overall propensity toward predicted stabilizing mutations. For instance, the substitution F61Y in CB initially exhibited a destabilizing ΔΔ*G* of −0.71 kcal/mol, but when collectively assessed in conjunction with S-21Y or H-F21Y (see Figure 8), the global stability incrementally improved to 0.62 and 0.6 kcal/mol, respectively (Table 2).^23^ This result not only corroborates the detected variants but also suggests that these substitutions may stem from a common uncharacterized precursor.

### Molecular Insights into Venom Toxin Diversification

Inter- and intraspecific divergence in *Crotalus sp.* venom composition may arise from various mechanisms, including transcriptional modulation^69,70^ and post-transcriptional regulation involving micro ribonucleic acids (miRNA).^71^ Rather than a simplistic compensation mechanism at the protein-family level, variation in *Crotalus sp*. venom composition implies a fine-tuned expression profile regulation, arising from locus-specific transcriptional modulation.^72^ The reported ontogenetic modulation translates into varying levels of major *Crotalus sp.* toxins, such as PLA_2_, SVSP, SVMPs, and crotamine at different developmental stages.^23,70,71,73^ For instance, when analyzing congeneric Mexican rattlesnakes, alterations in miRNA distribution were hypothesized to simultaneously silence the translation of neurotoxic heterodimeric PLA_2_ while up-regulating SVMPs expression.^71^ This dual transcriptional regulation explains the existence of divergent venom phenotypes responsible for the venom dichotomy between crotoxin-rich (neurotoxic) and SVMP-rich (hemorrhagic) venoms among rattlesnakes.^23,70,71,73^ Furthermore, despite the similar total levels of toxin classes when comparing *C. adamanteus* ^69^ and *C. s. simus* ^73^ adults and newborn/juvenile transcriptomes, featured paralogs differed substantially, highlighting phenotypical divergences at the proteoform level, thus corroborating the variation found in specific toxin proteoform abundances.^23^

Another possible mechanism, underlying shifts in crotamine content between males and females, is the variation in gene copy numbers, which has been shown to correlate with the abundance of this toxin in the venom.^23,74^ Interestingly, while rattlesnakes have been traditionally classified as crotamine positive or negative,^14,63^ this toxin was detected in all individuals in our proteomic analysis (). This result diverges from previous studies analyzing crotamine detection in *C. durissus* rattlesnake venom, which consider Cdt as mostly crotamine-negative.^13,15^ A possible explanation for crotamine detection in all specimens is the presence of polymorphic myotoxins (1–4), as the sequence heterogeneity among these peptides relies on single amino acid residues,^75^ which can be masked by enzymatic digestion at polybasic sites, thereby contributing to crotamine detection in proteomic analysis.^23^ Moreover, mapping crotamine *loci* on chromosome 2 of multiple individuals^74,76^ amplified crotamine and crotamine related genes (crotasin), but not myotoxin sequences,^75^ suggesting that other *loci* may be involved in myotoxin expression. This result suggests that proteomic determinations may be more suitable for the detection of these small polybasic myotoxic peptides than traditional assays.^63,77^ Nonetheless, the observation that crotamine-positive Cdt individuals present lower levels of crotoxin and metalloproteinases^13,60^ aligns with the lower abundance of crotoxin and MAD-3a in males, which exhibit a higher abundance of crotamine, further denoting a distinguishable sexual-based proteomic profile.^23^ Hence, snake venom sex shall also be relevant in determining snake venom complexity and should be taken into consideration when formulating an efficient crotalic antivenom.^23^ Regarding changes in other minor toxic components, studies on various snake species, including *C. adamanteus*,^69^ *C. atrox* ^78^ and *C. s. simus*,^79^ have revealed compositional shifts of BPP/C-type natriuretic peptides, CTL, NGF, BPP, LAAO, PDE, and CRISP when comparing developmental stages.^23^ However, these less abundant components are unlikely to significantly impact antivenom production and snakebite management.^12,78^

Recent advances in global mass spectrometry analysis have provided a powerful tool for conducting large-scale and in-depth profiling of protein variants,^23^ allowing for a precise and comprehensive characterization of diverse proteoforms.^21,53^ In this context, genetic diversification emerges as a key driver of the emergence of novel coding toxins within the venom polygenic trait.^3,4^ The genome’s abundance of structural and functional paralogs provides a selective advantage, particularly acting in synergy, as opposed to the traditional view of single-gene optimization.^6^ In contrast to the overdominant role of directional positive selection, where alleles with high fitness become fixed, emerging perspectives suggest the presence of distinct coevolutionary forces that shape multigene families.^23,80^ Evidence supports the involvement of balancing selection in maintaining allelic diversity, potentially driven by the advantages conferred by heterozygosity.^8,80^

Crotalinae venom toxins belong to multigene families that evolve through gene recruitment, extensive duplication events, accumulation of mutations, and retention of paralogous genes.^3,23,56,81^ Remarkably, highly abundant proteins experience stronger positive selection and relaxed selective constraints, leading to the accumulation of mutations and the expansion of phenotypic space.^5,23^ SVMPs provide valuable insights into molecular scaffold diversity originating from multiple gene retention, repeatedly punctuated by complete domain loss and adaptive evolution of surface-exposed amino acid residues, ultimately contributing to the consistent formation of distinct multidomain topologies.^23^ Similarly, a single large effect variation caused by a proline to serine substitution triggered a proteolysis cascade to generate the mature acidic Mojave toxin (PLA_2_), exposing the hydrophobic interface present in the ancestral protein and enabling the heterodimeric acidic-basic subunit interaction.^56^ This single variation in the acidic subunit was followed by a burst of positive and purifying selection that led to the loss of acyl-hydrolase activity, and acquisition of neurotoxic profile of rattlesnake’s venom^82^

Furthermore, secondary and minor proteins such as CRISP,^83^ CTL^84^ and VEGF,^85^ which exhibit lower levels of duplication, have accumulated mutations and expanded their functional repertoire while relatively preserving their ancestral molecular scaffold.^23,83–85^ Our variant analysis provides additional support for previous findings regarding the presence of extensively uncharacterized crotoxin proteoforms in Cdt venom.^15^ In addition, it further expands the variability discussion to less abundant, yet prominent multilocus toxin families, such as SVSPs and VEGF.^23^

The diversification of snake venom amino acid sequences has expanded into multigene families, driven by a stable molecular core of ancestral proteins.^86^ Snake venom toxins, including crotoxin, VEGF, and SVSPs, have been shown to originate from extensively cysteine-rich proteins that have undergone accelerated evolution, benefiting the stability provided by cysteine connectivity and enabling mutations toward functional diversification.^23,86^ Characterizing the molecular consequences of single amino acid variants on protein stability is essential for interpretation of the impact on protein structure, function, interactions, and phenotype.^42^ The detected variants did not occur in primary structural features, such as the predicted catalytical pocket of thrombin-like enzymes, heparin binding domain/receptor binding loops of VEGF, and key contact residues of crotoxin.^23^ Although distinct residue variations may completely abolish biological effects of snake venom toxins,^87^ the results underscore the conservation of structural features in evolutionary related proteins, which are minimally affected by variations, denoting that new foldings are unlikely to emerge during protein evolution.^23,88^

## Limitations

This study has some limitations. The sample size (*n* = 5 per sex) constrains the generalizability of the observed sex-based differences, although to our knowledge it represents the largest individual-level TMT-based quantitative proteomic analysis of Cdt venom to date. Age and geographic origin were not controlled as stratification variables, and their potential confounding effects on venom composition cannot be excluded. To ensure comparable captivity conditions across specimens, only individuals maintained in captivity for at least three years prior to venom extraction were included, following a criterion previously adopted for Cdt venom studies.^15^ Future studies with larger, geographically matched cohorts may disentangle the relative contributions of sex, ontogeny, and geographic origin to Cdt venom variability.

## Concluding Remarks

This study provided an integrative view of *C. d. terrificus* venom variability through the identification of novel peptide sequences, processing of toxins at noncanonical sites, sexual dimorphism, and analysis of toxin variants with conserved predicted three-dimensional structures. Although complementary proteogenomic approaches connecting the genotype, expression and phenotype are essential for comprehensive characterization of venom proteoforms, bottom-up proteomics and peptidomics have proven to be valuable tools for high-throughput detection of variants. Despite the progress made, several questions regarding Cdt venom remain unanswered. For instance, how are the multiple variants organized at the transcript and gene levels? Does this variability impact the assembly of the crotoxin subunits? Moreover, do these variants possess distinct phenotypic and pharmacological characteristics? Addressing these questions would provide valuable insights into the intricate nature of Cdt venom and contribute to the understanding of its biological and medical significance.

## Supplementary Material







## Data Availability

The mass spectrometry data sets generated in this study are publicly available through the ProteomeXchange Consortium via the PRIDE repository^38^ under accession numbers PXD040657 (peptidomics), PXD040735 (DIA proteomics), and PXD044419 (DDA proteomics).

## Notes

Figure S1. Electrophoretic profile of individual venom samples. Figure S2. Structural illustration of the phospholipase A_2_ homologue Crotoxin Acid Subunit (CA). Figures S3–S15. *C. d. terrificus* MS and MS/MS variant spectra. (PDF)
All quantitative data is provided as raw values, prior to imputation and normalization. Table S1. Biometric data of the *C. d. terrificus* specimens used for venom extraction. Table S2. *C. d. terrificus* venom peptides identified by LC-MS/MS analysis and database search. Table S3. *C. d. terrificus* novel venom peptides identified by *de novo* sequencing of LC-MS/MS data. Table S4. Quantitative proteomics of *C. d. terrificus* venom by DDA-TMT. Table S5. Validation of amino acid variation and quantitation of assigned proteoforms. (XLSX)
R Script. Sampling locality map for *Crotalus durissus terrificus* specimens written in R. (TXT)

## Abbreviations

ACN: acetonitrile
ACE: angiotensin-converting enzyme
AGC: automatic gain control
BPP: bradykinin-potentiating peptide
CA: crotoxin acidic subunit
CB: crotoxin basic subunit
Cdt: *Crotalus durissus terrificus*
CEUA: Ethics Committee on the Use of Animals
CRISP: cysteine-rich secretory protein
CTL: C-type lectin
CNP: C-type natriuretic peptide
DDA: data-dependent acquisition
DIA: data-independent acquisition
DTT: dithiothreitol
EDTA: ethylenediaminetetraacetic acid
FA: formic acid
FC: *log*_2_ ratio
FDR: false discovery rate
GIS: global internal standard
HDMSE: high-definition MSE
HPLC/UPLC: high-/ultraperformance liquid chromatography
LAAO: L-amino acid oxidase
LFQ: label-free quantification
LC-MS/MS: liquid chromatography–tandem mass spectrometry
MS: mass spectrometry
MS/MS: tandem mass spectrometry
MSE: DIA mode using alternating low/high collision energies
MW: molecular weight
PCA: principal component analysis
PDB: Protein Data Bank
PDE: phosphodiesterase; phospholipase A_2_
PLB: phospholipase B
PLA_2_: pLDDT Predicted Local Distance Difference Test
PMSF: phenylmethylsulfonyl fluoride
PTM: post-translational modification
rLys-C: recombinant Lys-C protease
SDS-PAGE: sodium dodecyl sulfate–polyacrylamide gel electrophoresis
SNP: single-nucleotide polymorphism
SVMP: snake venom metalloproteinase
SVSP: snake venom serine proteinase
TFA: trifluoroacetic acid
TMT: tandem mass tag
VEGF: vascular endothelial growth factor
ΔΔ*G*: change in Gibbs free energy
