# Supplementary material for: Sexual Dimorphism and Intraspecific Toxin Variation in Crotalus durissus terrificus Venom: Insights from Proteomics, Peptidomics, and Structural Modeling
Source: J Proteome Res. 2026 Sep 4;25(10):5230–45. doi: 10.1021/acs.jproteome.6c00275 (PMC13640431; doi:10.1021/acs.jproteome.6c00275)
Supplement: Supplementary file 1 [file pr6c00275_si_001.pdf]

Sexual Dimorphism and Intraspecific Toxin Variation in *Crotalus durissus terrificus* Venom: Insights from Proteomics, Peptidomics, and Structural Modelling

Jackson G. Miyamoto[1], Jackelinne Y. Hayashi[1], Giuseppe G. Figueirêdo Leite[2], Sávio S. Sant'Anna[3], Hang Fai Kwok[4], Richardt H. Valente[5], Anita M. Tanaka-Azevedo[3], Alexandre K. Tashima[1*]

[1]Department of Biochemistry, Escola Paulista de Medicina, Universidade Federal de São Paulo, São Paulo 04039-032, Brazil

[2]Laboratory of Mass Spectrometry Applied to Biomolecules, Escola Paulista de Medicina, Universidade Federal de São Paulo, São Paulo 04039-032, Brazil

[3]Division of Infectious Diseases, Department of Medicine, Escola Paulista de Medicina, Universidade Federal de São Paulo, São Paulo 04039-032, Brazil.

[4]Laboratory of Herpetology, Butantan Institute, São Paulo 05503-900, SP, Brazil.

[5]Department of Biomedical Sciences, Faculty of Health Sciences, Avenida de Universidade, University of Macau, 999078, Macau SAR

[6]Laboratory of Toxinology, Oswaldo Cruz Institute, Oswaldo Cruz Foundation, FIOCRUZ, Rio de Janeiro 21040-900, Brazil

*Corresponding Author: Alexandre Keiji Tashima, aktashima@unifesp.br

Table of contents

Supplementary Figures

Figure S1. Electrophoretic profile of individual venom samples.

Figure S2. Structural illustration of the phospholipase A_2 homolog Crotoxin Acid Subunit (CA).

Figures S3-S15. *C. d. terrificus* representative MS and MS/MS spectra of mutated peptides.

Supplementary Tables

Table S1. Biometric data of the *C. d. terrificus* specimens selected for venom extraction.

Table S2. *C. d. terrificus* venom peptides identified by LC-MS/MS analysis and database search.

Table S3. *C. d. terrificus* novel venom peptides identified by *de novo* sequencing of LC-MS/MS data.

Table S4. Quantitative proteomics of *C. d. terrificus* venom by DDA-TMT.

Table S5. Validation of mutations and quantification of assigned isoforms.

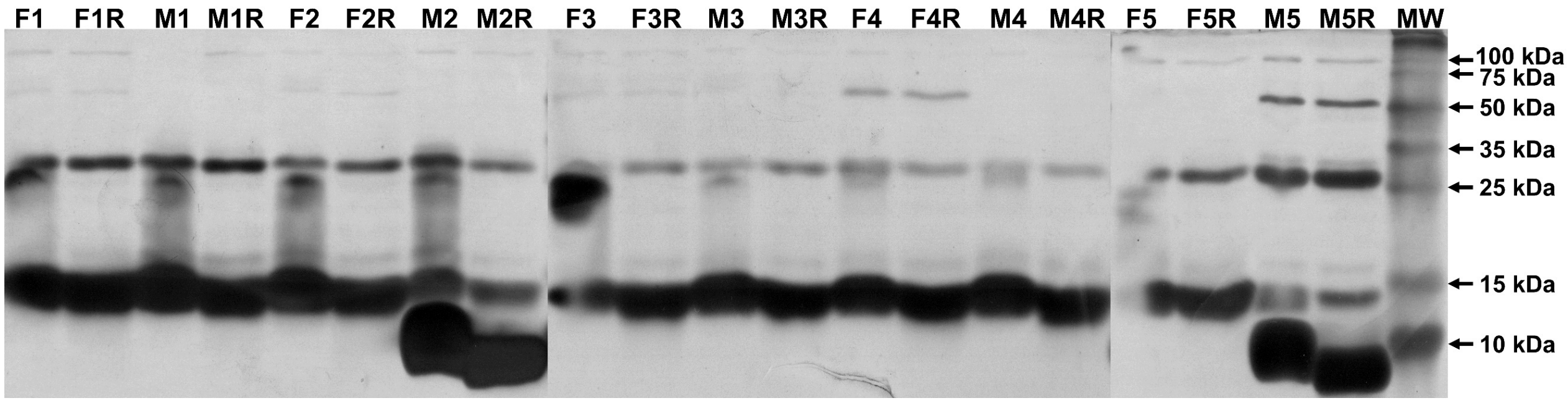

Figure S1. Electrophoretic profiles of individual male (M) and female (F) venom samples (15 µg) subjected to SDS-PAGE 15% under non-reducing and reducing (R) conditions and stained with 12 mM silver nitrate. MW: molecular weight marker (Broad Range Protein Molecular Weigh Markers – Promega).

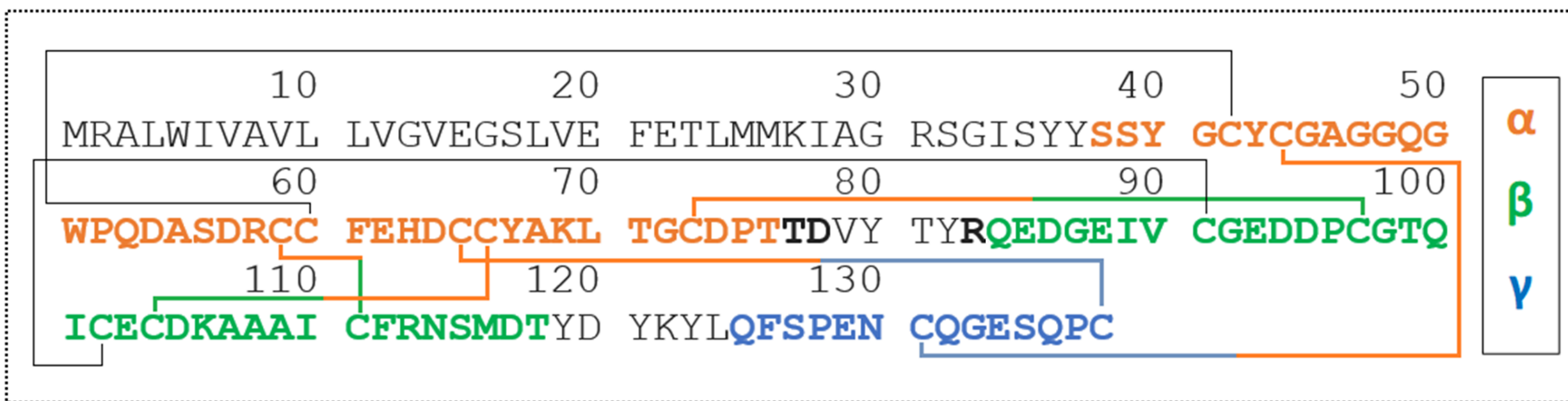

Figure S2. Primary structure of PLA_2 homolog Crotoxin Acid (CA) subunit. The three chains are displayed with distinct colors: α-chain (orange), β-chain (green), and γ-chain (blue). Variable extensions with experimental evidence are depicted in bold (black). Intra-chain cross-links are shown as solid lines, while inter-chain cross-links are colored according to the identity of the linked chain.

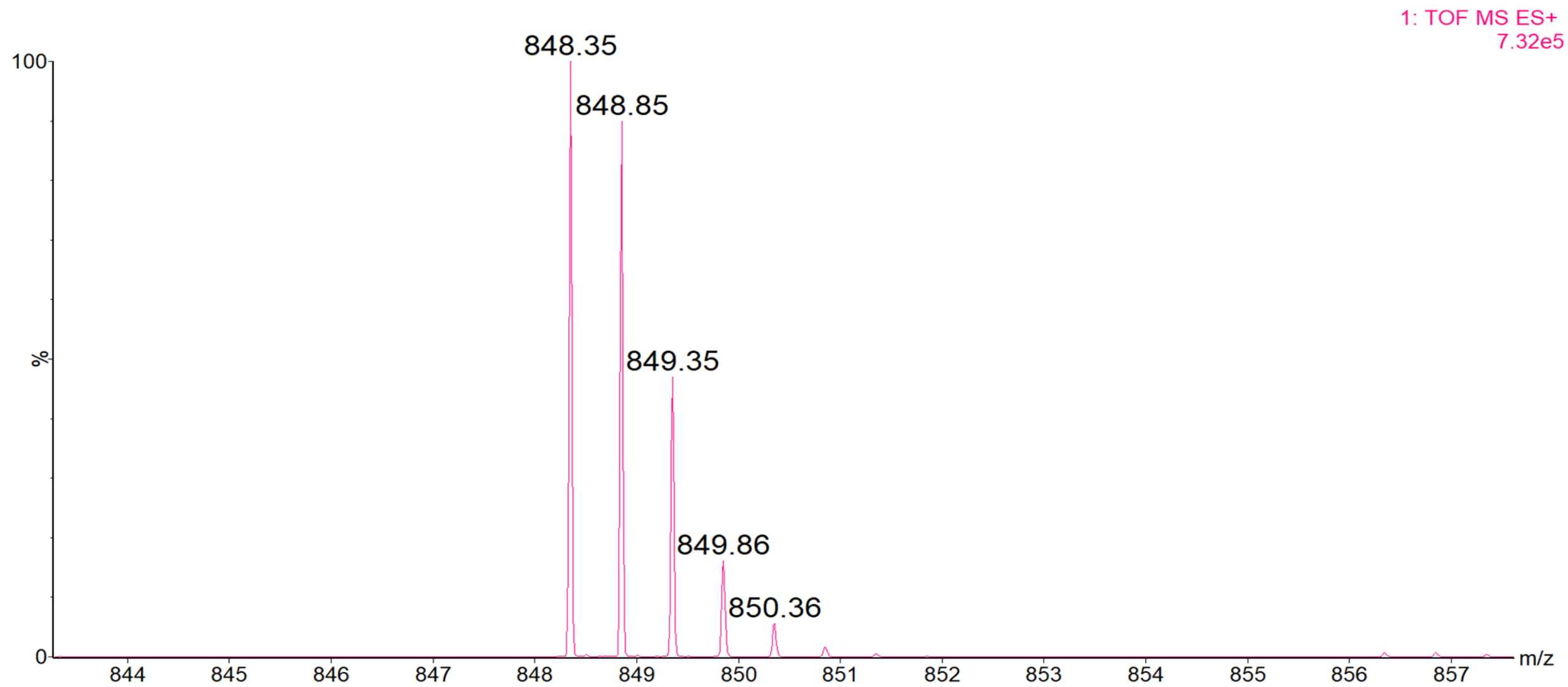

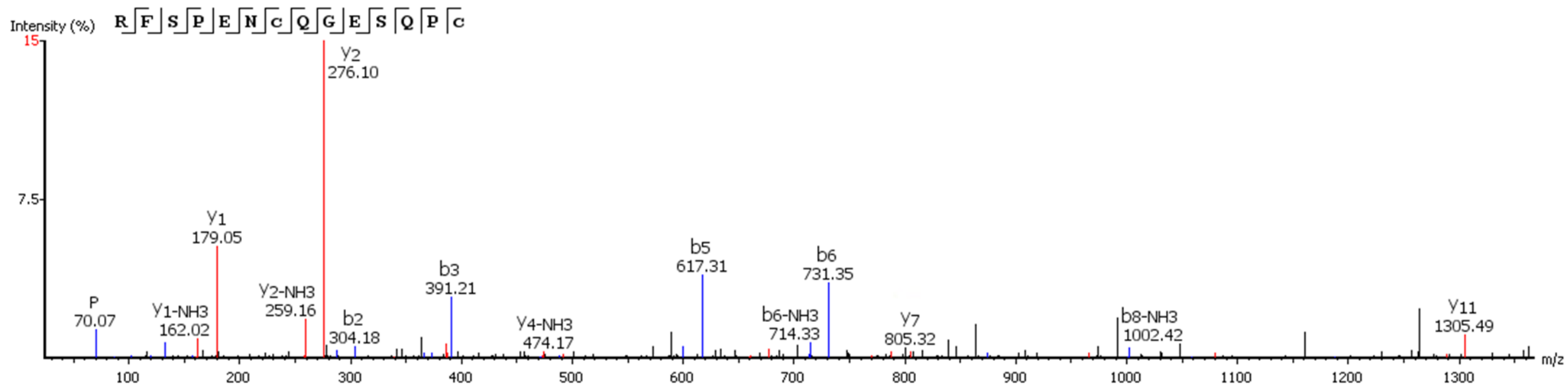

Ion Match

#	Immonium	b	b-H2O	b-NH3	b (2+)	Seq	y	y-H2O	y-NH3	y (2+)	#
1	129.11	157.11	139.10	140.08	79.05	R					14
2	120.08	304.18	286.17	287.15	152.59	F	1539.58	1521.57	1522.56	770.28	13
3	60.04	391.21	373.20	374.18	196.10	S	1392.52	1374.50	1375.49	696.76	12
4	70.07	488.27	470.26	471.23	244.63	P	1305.49	1287.47	1288.46	653.24	11
5	102.06	617.31	599.30	600.28	309.15	E	1208.43	1190.42	1191.40	604.72	10
6	87.06	731.35	713.34	714.33	366.15	N	1079.39	1061.38	1062.36	540.19	9
7	133.04	891.38	873.37	874.36	446.19	C(+57.02)	965.35	947.33	948.32	483.17	8
8	101.07	1019.44	1001.44	1002.42	510.22	Q	805.32	787.30	788.29	403.16	7
9	30.03	1076.46	1058.46	1059.43	538.73	G	677.26	659.24	660.23	339.14	6
10	102.06	1205.50	1187.50	1188.47	603.25	E	620.23	602.22	603.21	310.62	5
11	60.04	1292.53	1274.52	1275.51	646.77	S	491.20	473.18	474.17	246.10	4
12	101.07	1420.59	1402.58	1403.56	710.80	Q	404.16	386.15	387.14	202.58	3
13	70.07	1517.64	1499.63	1500.62	759.32	P	276.10	258.08	259.10	138.55	2
14	133.04					C(+57.02)	179.05	161.04	162.02	90.02	1

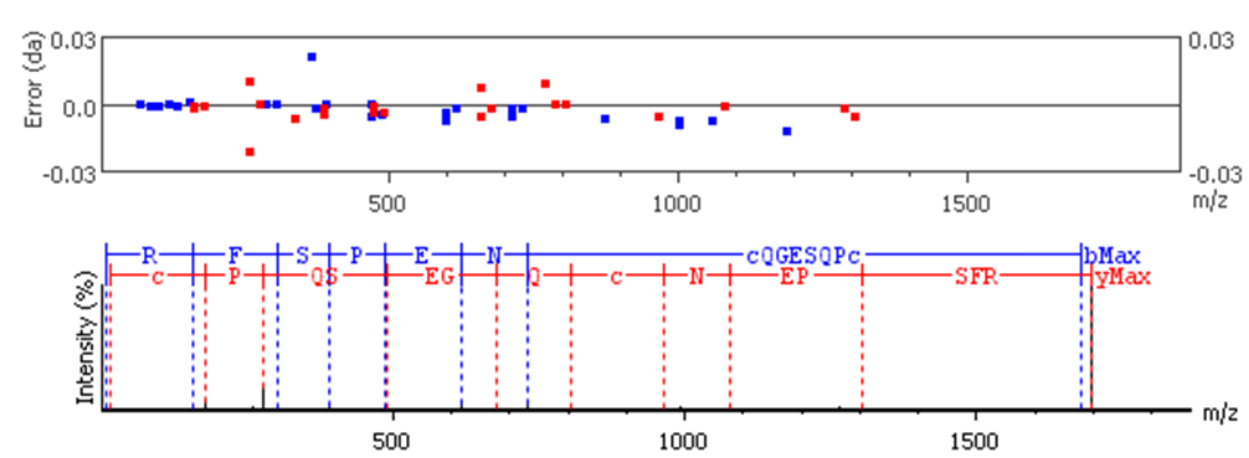

Figure S3. MS1 and MS2 spectra of Gln^{125} → Arg (+28.04 Da) residue substitution in PLA2 CA subunit, digested gamma chain peptide RFSPENCQGESQPC (nonmutated peptide: QFSPENCQGESQPC). Peptide observed at m/z 848.35^{+2}. Fragment ions assigned using PEAKS Studio X+ (BSI).

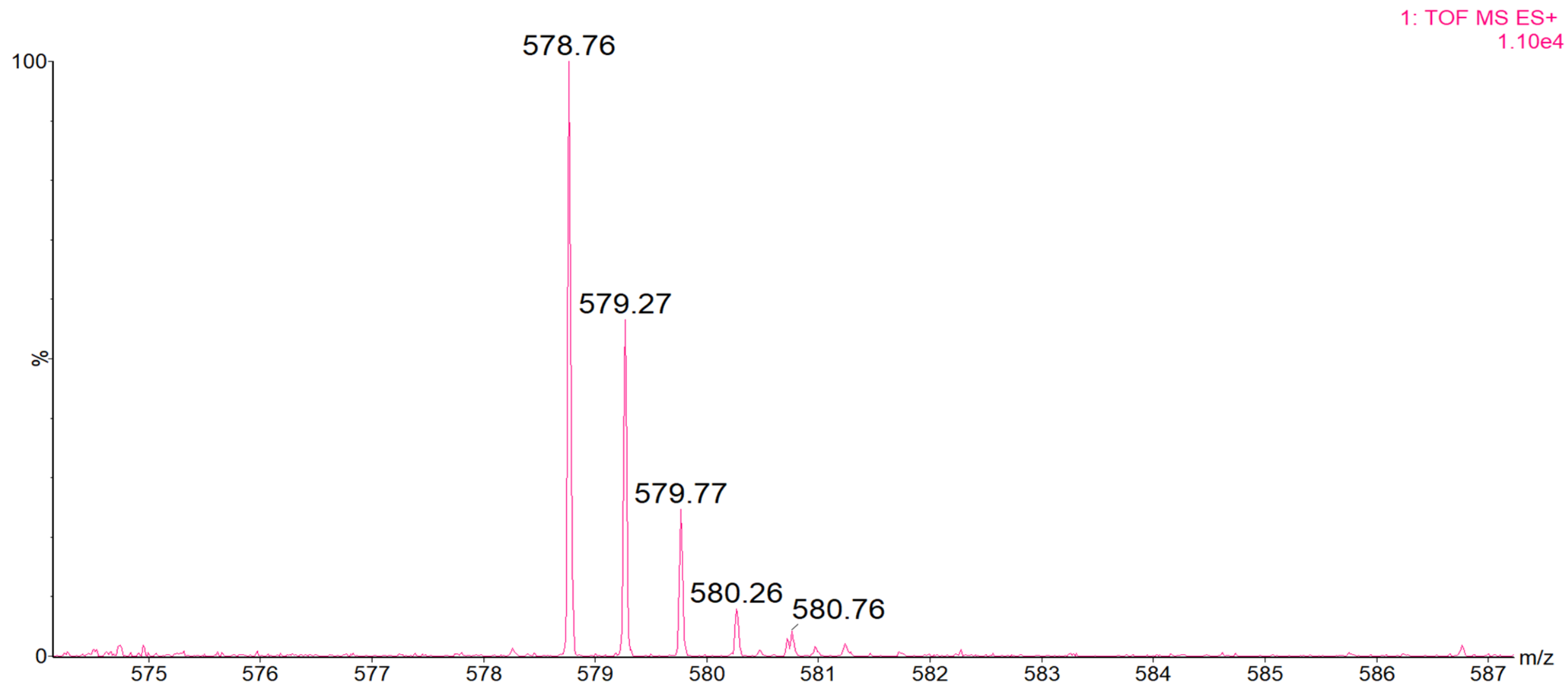

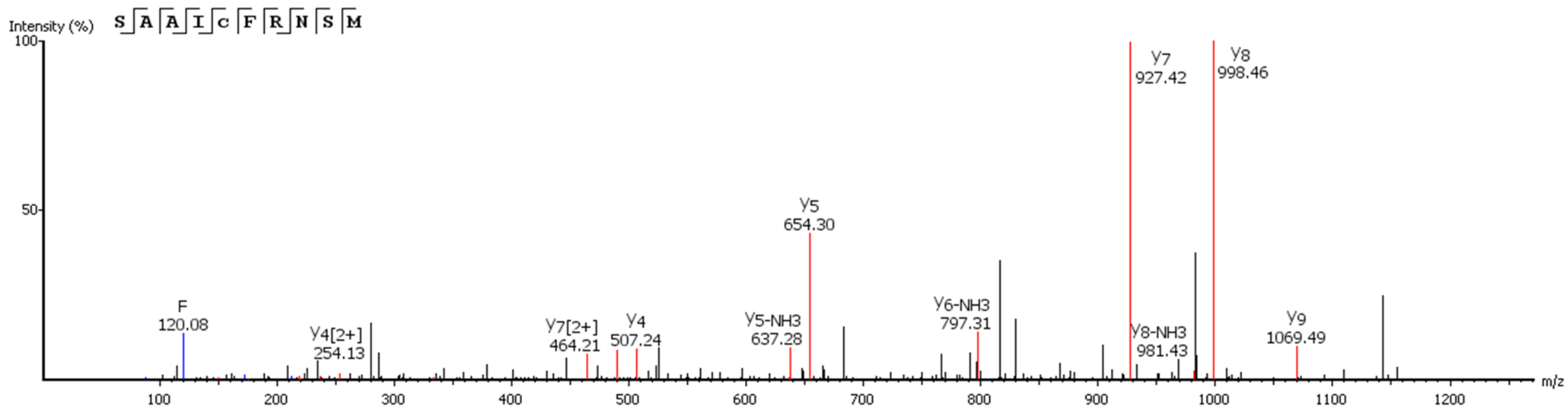

Ion Match

#	Immonium	b	b-H2O	b-NH3	b (2+)	Seq	y	y-H2O	y-NH3	y (2+)	#
1	60.04	88.02	70.03	71.01	44.52	S					10
2	44.05	159.08	141.07	142.05	80.04	A	1069.49	1051.48	1052.46	535.25	9
3	44.05	230.11	212.10	213.09	115.56	A	998.46	980.44	981.43	499.73	8
4	86.10	343.20	325.19	326.17	172.08	I	927.42	909.41	910.39	464.21	7
5	133.04	503.23	485.22	486.20	252.11	C(+57.02)	814.33	796.32	797.31	407.67	6
6	120.08	650.30	632.29	633.27	325.65	F	654.30	636.29	637.28	327.65	5
7	129.11	806.42	788.39	789.37	403.70	R	507.24	489.22	490.21	254.13	4
8	87.06	920.44	902.43	903.41	460.72	N	351.13	333.12	334.11	176.05	3
9	60.04	1007.47	989.46	990.45	504.24	S	237.10	219.07	220.07	119.05	2
10	104.05					M	150.06	132.05	133.03	75.53	1

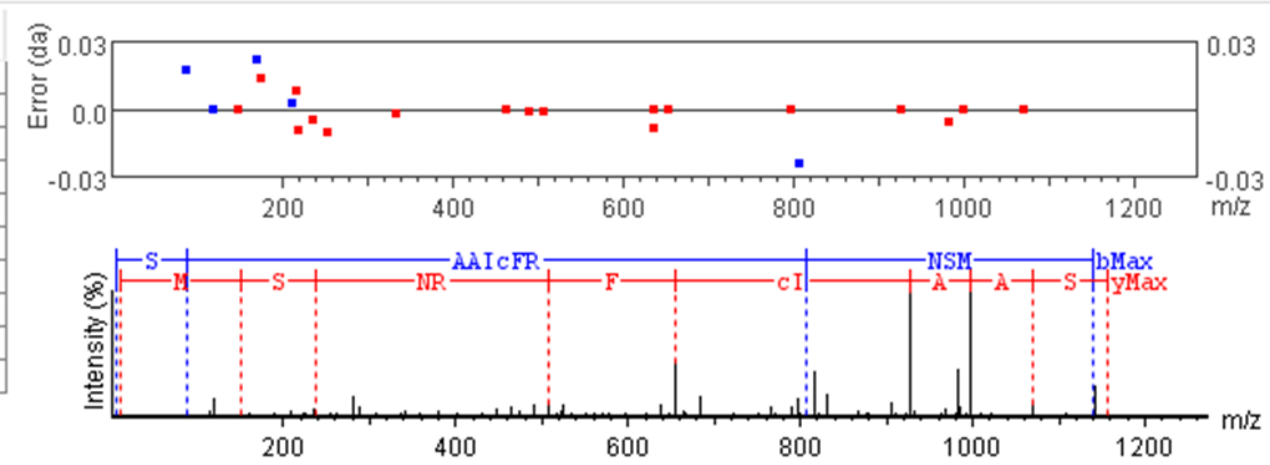

Figure S4. MS1 and MS2 spectra of Ala[107] → Ser (+15.99 Da) residue substitution in PLA2 CA subunit, beta chain peptide SAAICFRNSM (nonmutated peptide: AAAICFRNSMDT). Peptide observed at m/z 578.76^{+2}. Fragment ions assigned using PEAKS Studio X+ (BSI).

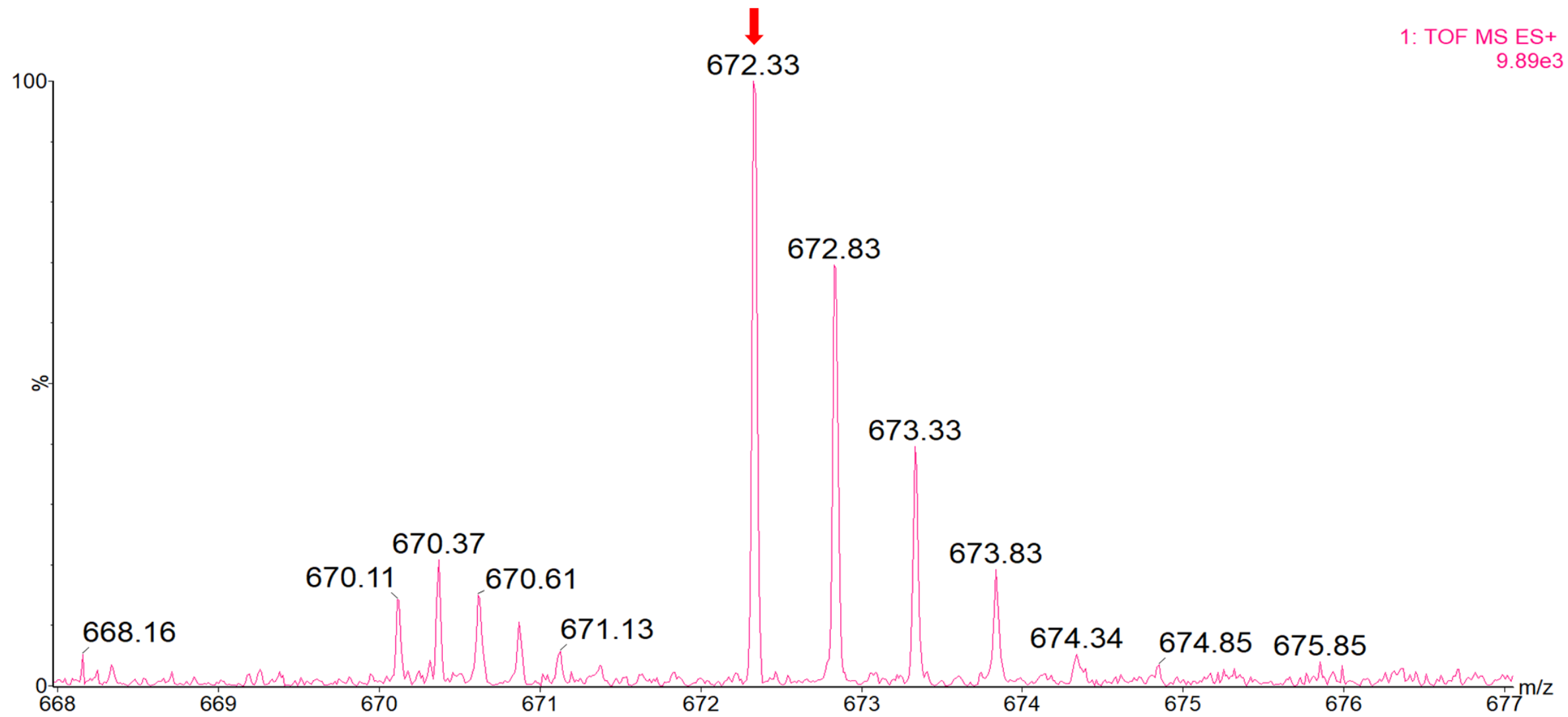

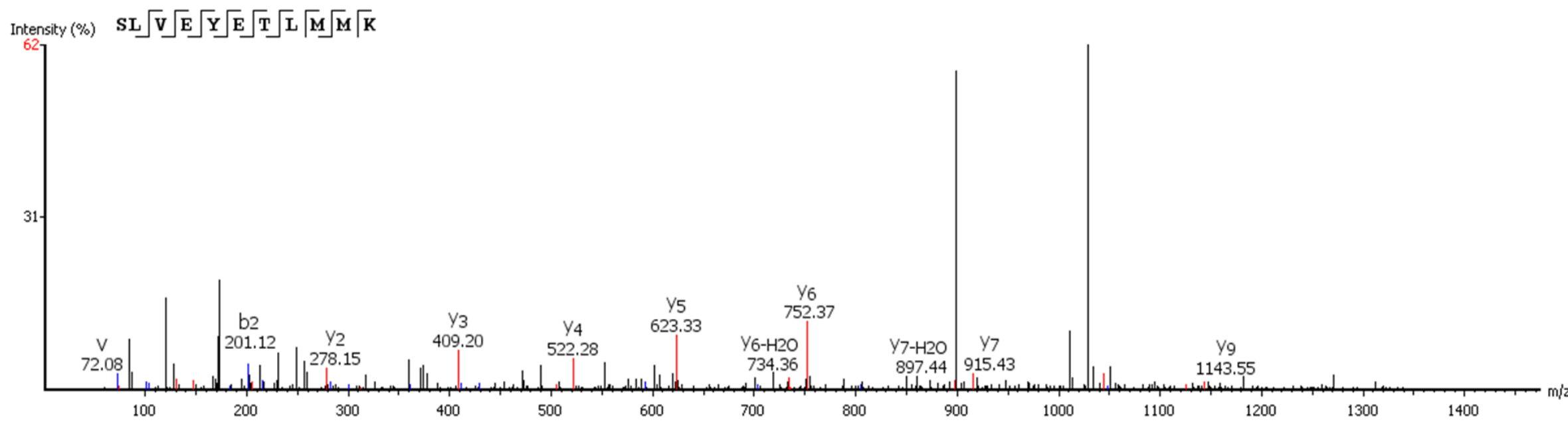

Ion Match

#	Immonium	b	b-H2O	b-NH3	b (2+)	Seq	y	y-H2O	y-NH3	y (2+)	#
1	60.04	88.04	70.03	71.01	44.52	S					11
2	86.10	201.12	183.12	184.10	101.07	L	1256.63	1238.62	1239.60	628.81	10
3	72.08	300.19	282.18	283.17	150.60	V	1143.55	1125.51	1126.52	572.27	9
4	102.06	429.24	411.20	412.21	215.14	E	1044.48	1026.46	1027.45	522.74	8
5	136.08	592.30	574.29	575.27	296.65	Y	915.43	897.44	898.40	458.22	7
6	102.06	721.34	703.34	704.31	361.15	E	752.37	734.36	735.34	376.68	6
7	74.06	822.39	804.37	805.36	411.69	T	623.33	605.31	606.30	312.16	5
8	86.10	935.47	917.46	918.45	468.24	L	522.28	504.27	505.23	261.64	4
9	104.06	1066.51	1048.51	1049.49	533.76	M	409.20	391.18	392.17	205.10	3
10	104.05	1197.55	1179.54	1180.53	599.28	M	278.15	260.14	261.13	139.58	2
11	101.11					K	147.11	129.10	130.08	74.06	1

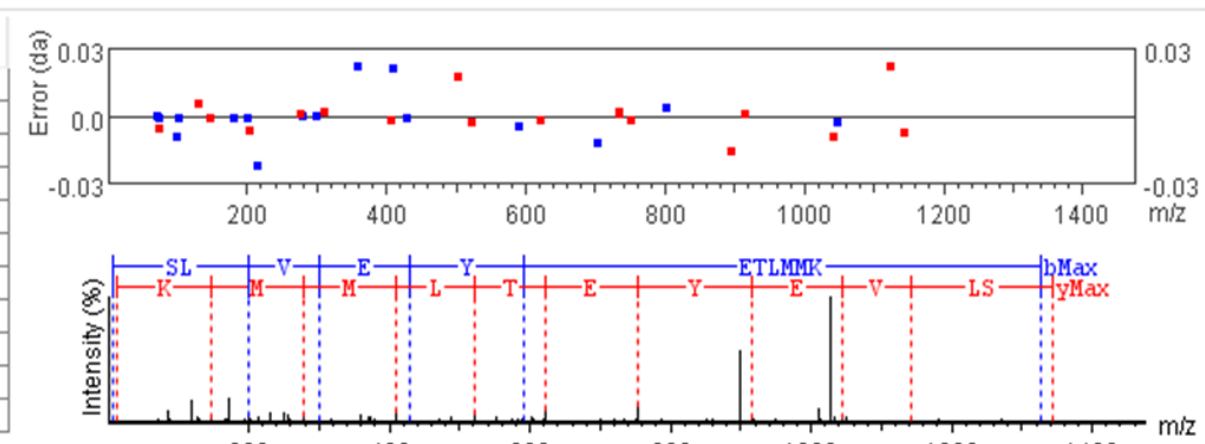

Figure S5. MS1 and MS2 spectra of Phe[21] → Tyr (+15.99 Da) residue substitution in PLA2 CA subunit, signal peptide SLVEYETLMMK (nonmutated peptide: SLVEFETLMMK). Peptide observed at m/z 672.33^{+2}. Fragment ions assigned using PEAKS Studio X+ (BSI).

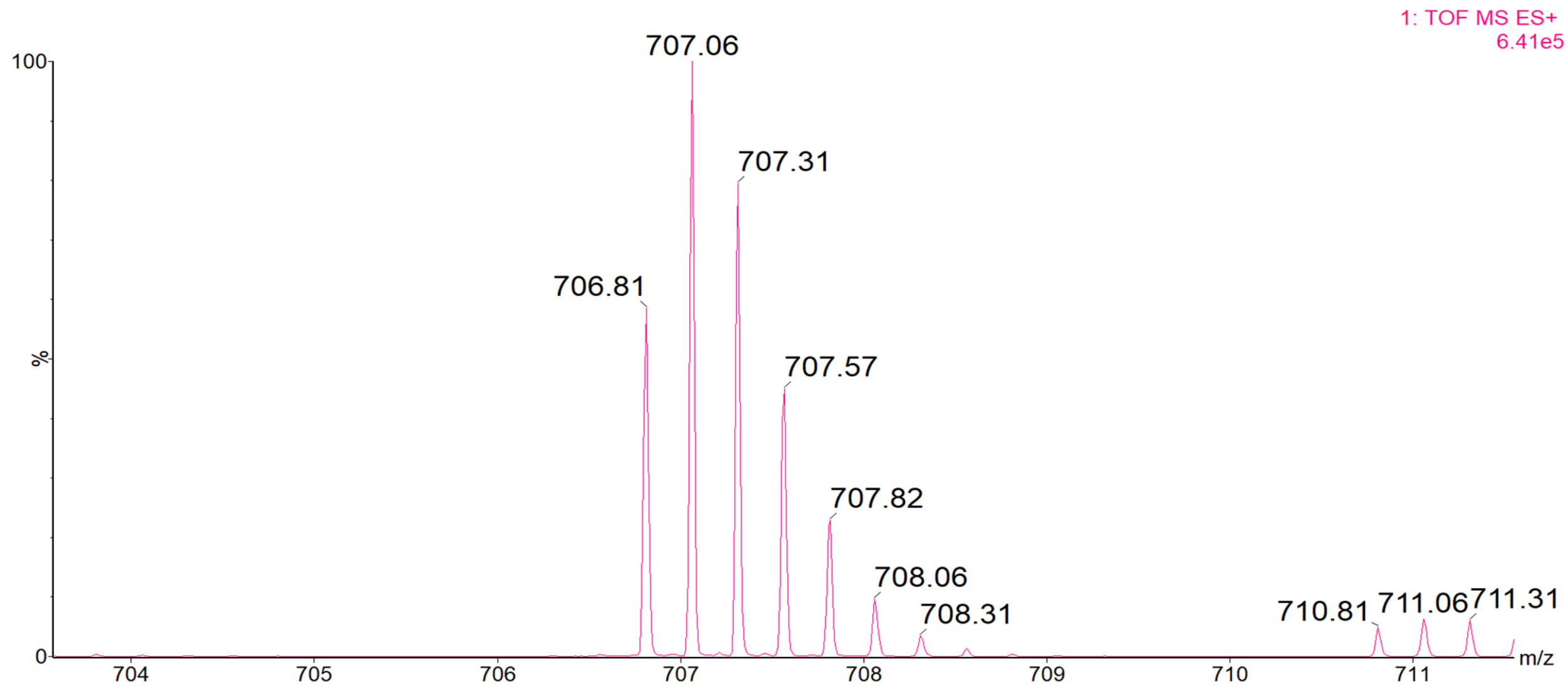

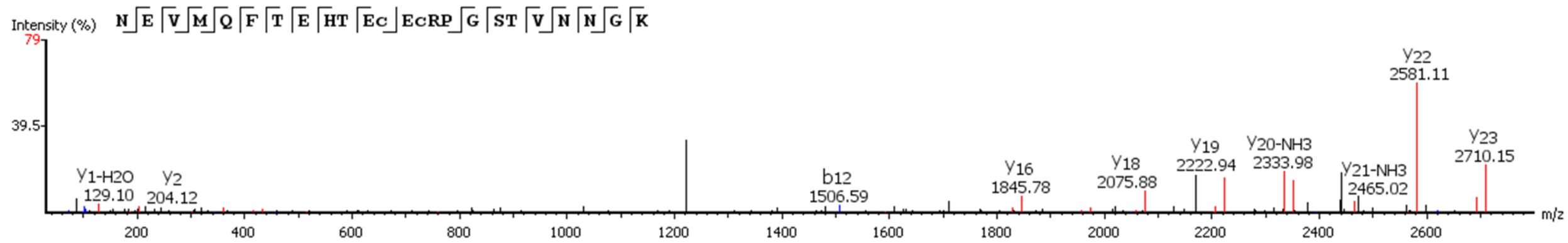

Ion Match

#	Immonium	b	b-H2O	b-NH3	b (2+)	Seq	y	y-H2O	y-NH3	y (2+)	#
1	87.06	115.05	97.04	98.05	58.03	N					24
2	102.06	244.09	226.08	227.07	122.55	E	2710.15	2692.15	2693.14	1355.58	23
3	72.08	343.17	325.15	326.13	172.08	V	2581.11	2563.11	2564.10	1291.06	22
4	104.06	474.20	456.19	457.19	237.60	M	2482.05	2464.04	2465.02	1241.53	21
5	101.07	602.26	584.25	585.23	301.63	Q	2351.00	2333.00	2333.98	1176.01	20
6	120.08	749.33	731.32	732.30	375.16	F	2222.94	2204.93	2205.93	1111.98	19
7	74.06	850.38	832.37	833.35	425.69	T	2075.88	2057.88	2058.85	1038.44	18
8	102.06	979.42	961.41	962.39	490.21	E	1974.83	1956.81	1957.81	987.92	17
9	110.07	1116.48	1098.47	1099.45	558.74	H	1845.78	1827.79	1828.77	923.40	16
10	74.06	1217.53	1199.52	1200.50	609.26	T	1708.74	1690.73	1691.71	854.87	15
11	102.06	1346.57	1328.56	1329.54	673.78	E	1607.69	1589.68	1590.65	804.35	14
12	133.04	1506.59	1488.59	1489.57	753.80	C(+57.02)	1478.65	1460.64	1461.62	739.82	13
13	102.06	1635.64	1617.63	1618.61	818.32	E	1318.62	1300.61	1301.59	659.81	12
14	133.04	1795.67	1777.66	1778.65	898.34	C(+57.02)	1189.57	1171.56	1172.55	595.29	11
15	129.10	1951.77	1933.76	1934.75	976.39	R	1029.54	1011.53	1012.52	515.27	10
16	70.07	2048.84	2030.82	2031.80	1024.91	P	873.44	855.43	856.42	437.22	9
17	30.03	2105.85	2087.84	2088.82	1053.42	G	776.39	758.39	759.36	388.69	8
18	60.04	2192.88	2174.87	2175.85	1096.94	S	719.37	701.36	702.34	360.16	7
19	74.06	2293.93	2275.92	2276.90	1147.46	T	632.34	614.33	615.31	316.67	6
20	72.08	2393.00	2374.99	2375.97	1197.00	V	531.29	513.28	514.26	266.14	5
21	87.06	2507.04	2489.03	2490.01	1254.02	N	432.21	414.21	415.19	216.61	4
22	87.06	2621.10	2603.07	2604.05	1311.04	N	318.18	300.17	301.15	159.59	3
23	30.03	2678.10	2660.09	2661.08	1339.55	G	204.12	186.13	187.11	102.57	2
24	101.11					K	147.11	129.10	130.09	74.06	1

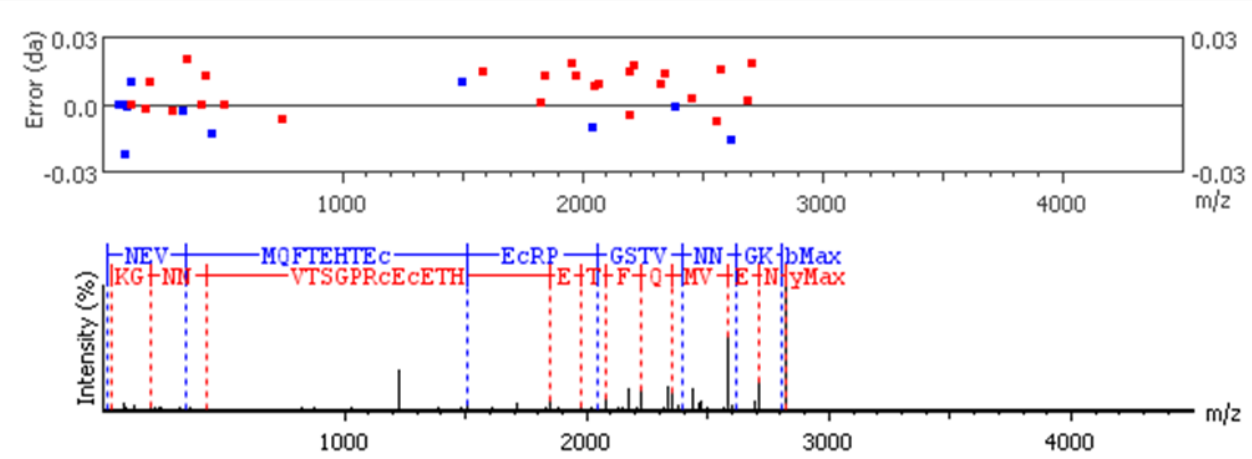

Figure S6. MS1 and MS2 spectra of Ile^{104} → Asn (+0.96 Da) residue substitution in CdtVEGF4 natural peptide NEVMQFTEHTECECRPGSTVNNGK (nonmutated peptide: IEVMQFTEHTECECRPGSTVNNGK). Peptide observed at m/z 786.81^{+4}. Fragment ions assigned using PEAKS Studio X+ (BSI).

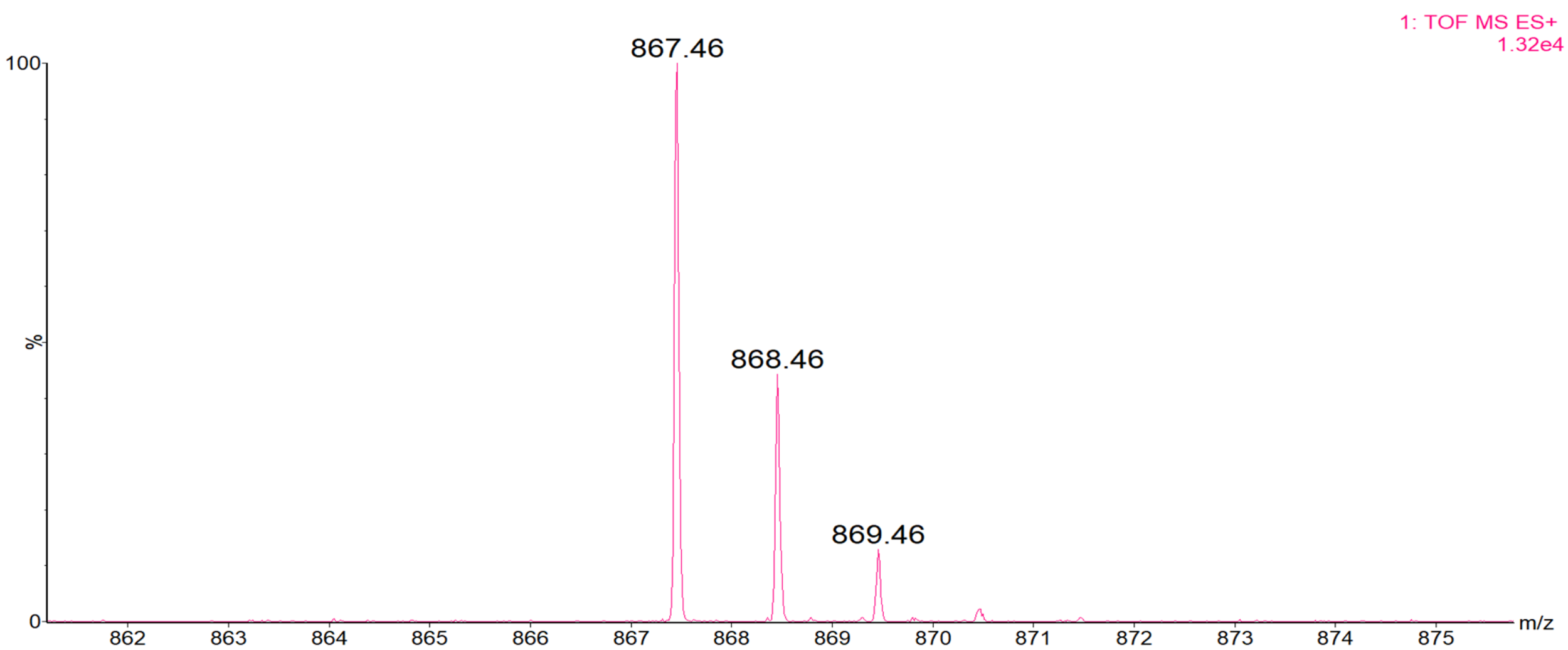

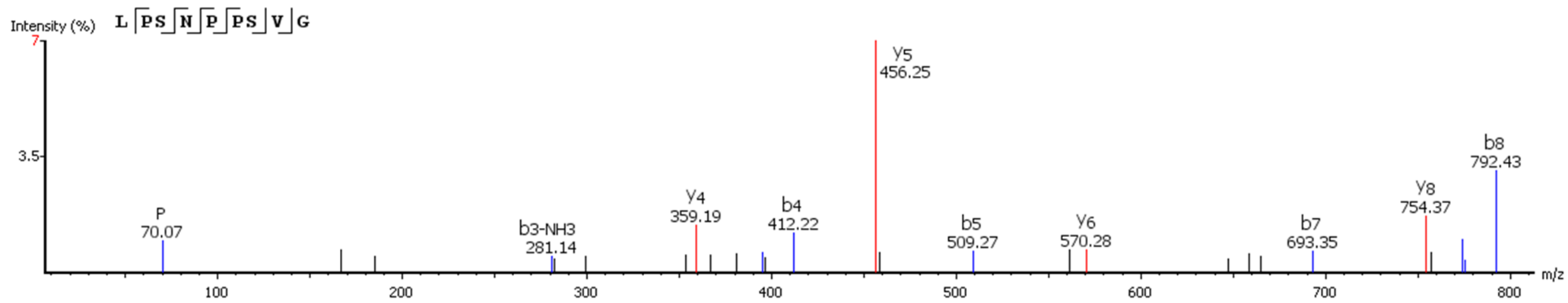

Ion Match

#	Immonium	b	b-H2O	b-NH3	b (2+)	Seq	y	y-H2O	y-NH3	y (2+)	#
1	86.10	114.09	96.08	97.06	57.55	L					9
2	70.07	211.14	193.13	194.12	106.07	P	754.37	736.36	737.35	377.69	8
3	60.04	298.18	280.17	281.14	149.59	S	657.32	639.31	640.29	329.16	7
4	87.06	412.22	394.21	395.19	206.61	N	570.28	552.28	553.26	285.64	6
5	70.07	509.27	491.26	492.25	255.14	P	456.25	438.23	439.22	228.62	5
6	70.07	606.33	588.31	589.30	303.66	P	359.19	341.18	342.17	180.10	4
7	60.04	693.35	675.35	676.33	347.18	S	262.14	244.13	245.11	131.57	3
8	72.08	792.43	774.42	775.40	396.71	V	175.11	157.10	158.08	88.05	2
9	30.03					G	76.04	58.03	59.01	38.52	1

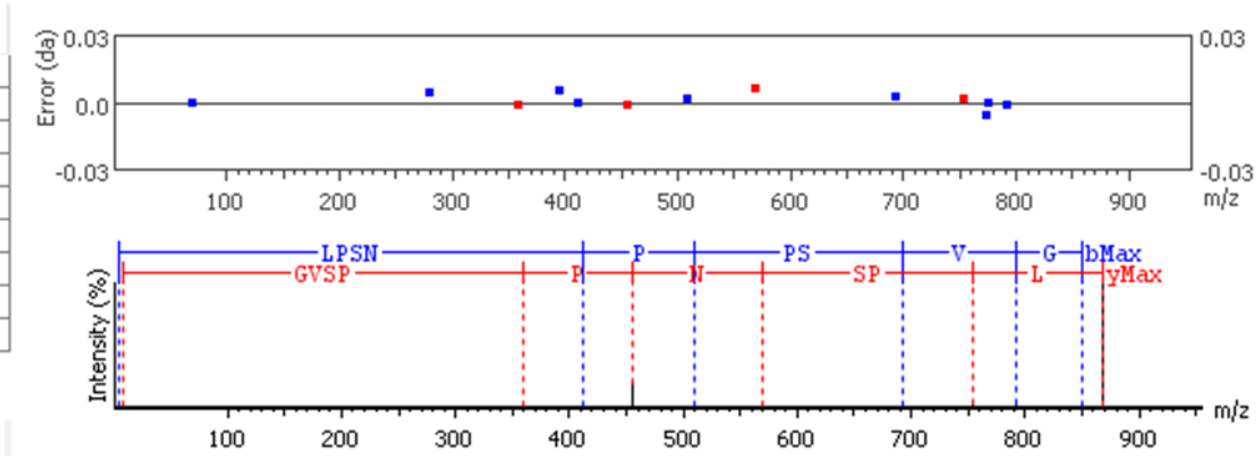

Figure S7. MS1 and MS2 spectra of Ser136 → Asn (+27.01 Da) residue substitution in gyroxin natural peptide LPSNPPSVG (nonmutated peptide: LPSSPPSVG). Peptide observed at m/z 867.46^{+1}. Fragment ions assigned using PEAKS Studio X+ (BSI).

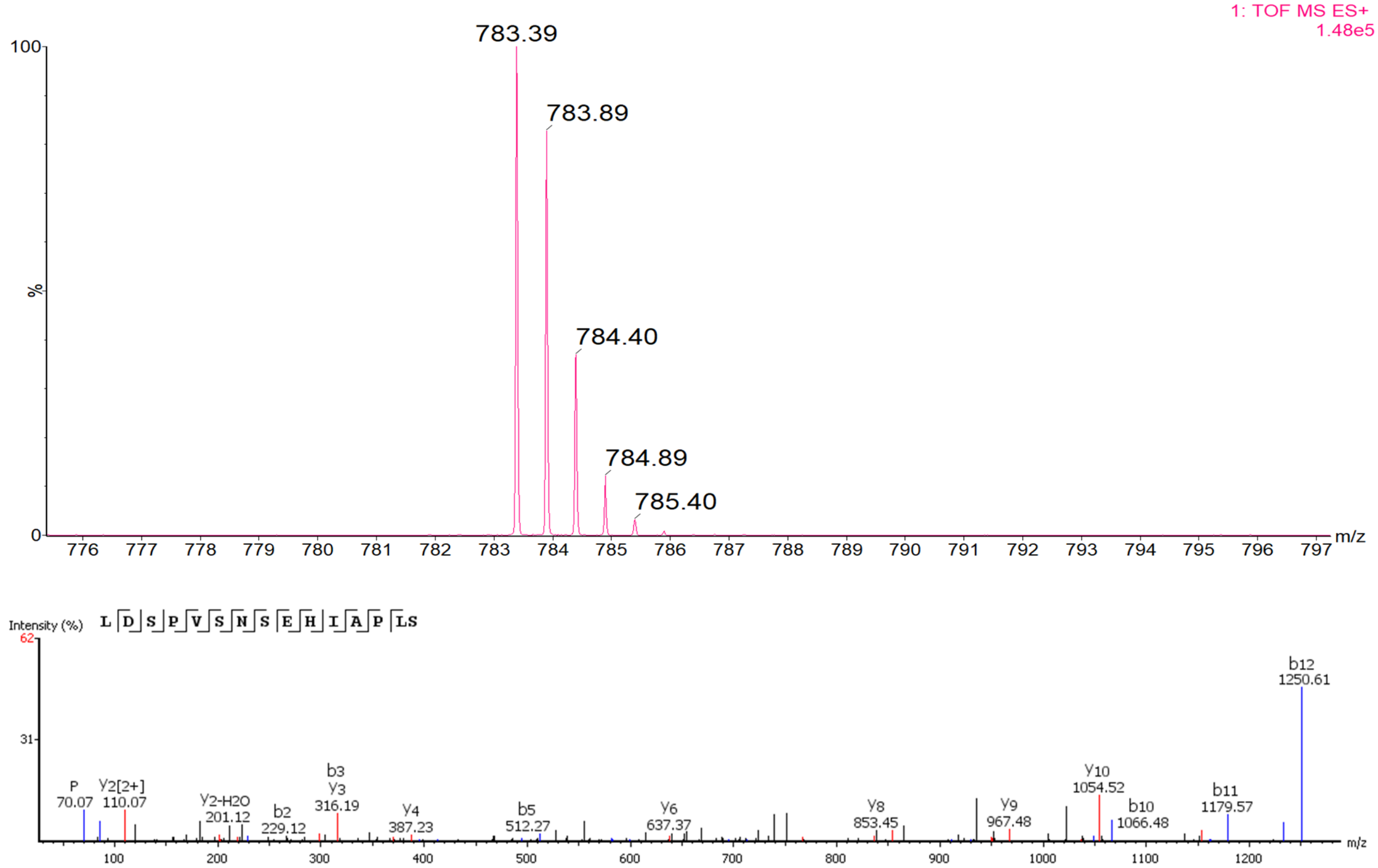

Ion Match

#	Immonium	b	b-H2O	b-NH3	b (2+)	Seq	y	y-H2O	y-NH3	y (2+)	#
1	86.10	114.09	96.08	97.06	57.55	L					15
2	88.04	229.12	211.11	212.09	115.06	D	1452.70	1434.69	1435.67	726.85	14
3	60.04	316.15	298.14	299.12	158.58	S	1337.67	1319.66	1320.64	669.33	13
4	70.07	413.20	395.19	396.18	207.10	P	1250.64	1232.63	1233.61	625.82	12
5	72.08	512.27	494.27	495.25	256.64	V	1153.58	1135.57	1136.56	577.29	11
6	60.04	599.29	581.27	582.29	300.15	S	1054.52	1036.51	1037.49	527.76	10
7	87.06	713.35	695.34	696.32	357.17	N	967.48	949.48	950.47	484.24	9
8	60.04	800.38	782.37	783.35	400.69	S	853.45	835.43	836.42	427.22	8
9	102.06	929.43	911.42	912.39	465.21	E	766.40	748.40	749.38	383.70	7
10	110.07	1066.48	1048.47	1049.45	533.74	H	637.37	619.36	620.34	319.18	6
11	86.10	1179.57	1161.57	1162.56	590.28	I	500.31	482.30	483.28	250.65	5
12	44.05	1250.61	1232.59	1233.57	625.80	A	387.23	369.21	370.20	194.11	4
13	70.07	1347.65	1329.64	1330.63	674.33	P	316.19	298.18	299.16	158.59	3
14	86.10	1460.74	1442.73	1443.71	730.87	L	219.14	201.12	202.08	110.07	2
15	60.04					S	106.05	88.04	89.02	53.52	1

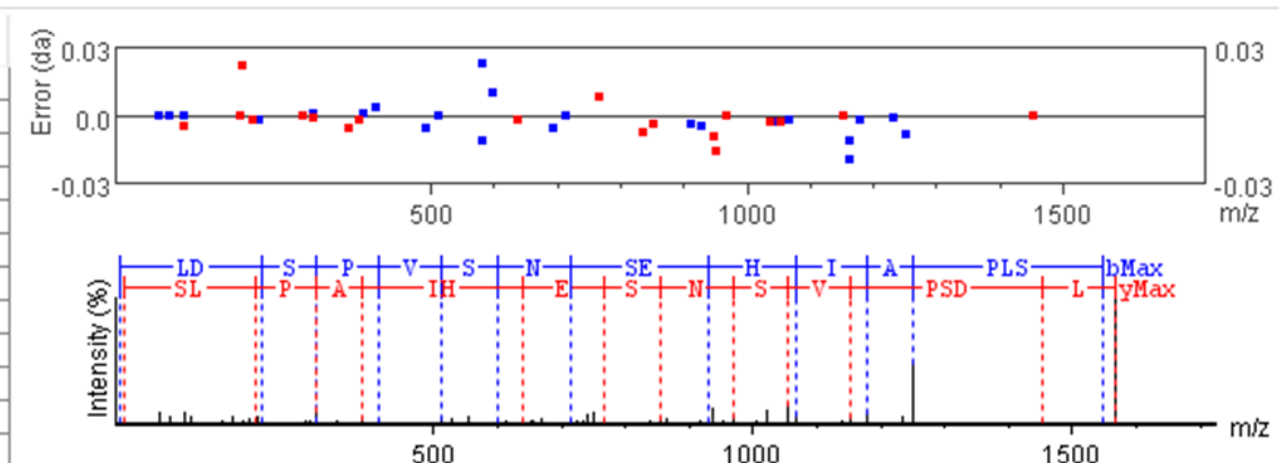

Figure S8. MS1 and MS2 spectra of Asn[119] → Asp (+0.98 Da), Lys[120] → Ser (-41.06 Da), and Tyr[124] → Asn (-49.02 Da) residue substitutions in gyroxin natural peptide LDSPVSNSEHIAPLS (nonmutated peptide: LNKPVSYSEHIAPLS). Peptide observed at m/z 783.39^{+2}. Fragment ions assigned using PEAKS Studio X+ (BSI).

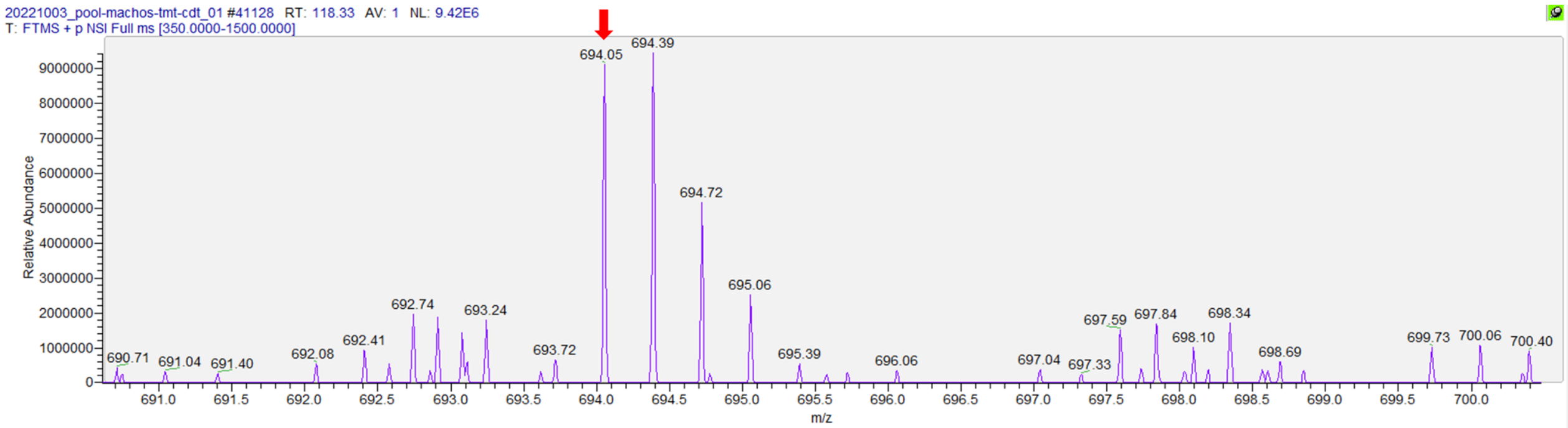

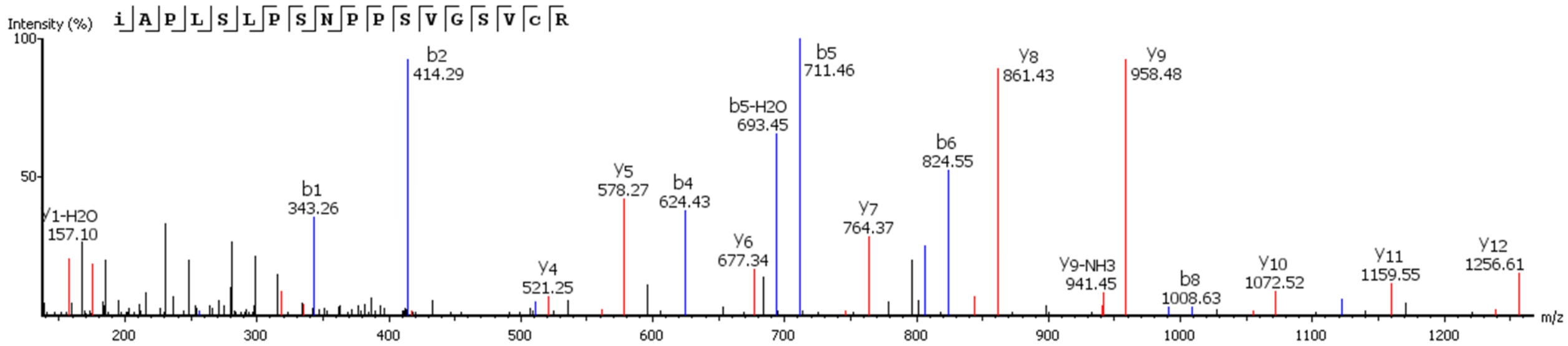

Ion Match

#	Immonium	b	b-H2O	b-NH3	b (2+)	Seq	y	y-H2O	y-NH3	y (2+)	#
1	315.26	343.26	325.24	326.23	172.13	I(+229.16)					18
2	44.05	414.29	396.28	397.26	207.65	A	1737.90	1719.88	1720.87	869.45	17
3	70.07	511.35	493.33	494.32	256.18	P	1666.86	1648.85	1649.83	833.93	16
4	86.10	624.43	606.42	607.40	312.71	L	1569.81	1551.79	1552.78	785.40	15
5	60.04	711.46	693.45	694.43	356.23	S	1456.72	1438.71	1439.69	728.86	14
6	86.10	824.55	806.53	807.52	412.77	L	1369.69	1351.68	1352.66	685.34	13
7	70.07	921.60	903.59	904.57	461.30	P	1256.61	1238.59	1239.58	628.80	12
8	60.04	1008.63	990.62	991.60	504.81	S	1159.55	1141.54	1142.53	580.28	11
9	87.06	1122.67	1104.66	1105.65	561.84	N	1072.52	1054.51	1055.49	536.76	10
10	70.07	1219.73	1201.71	1202.70	610.36	P	958.48	940.47	941.45	479.74	9
11	70.07	1316.78	1298.77	1299.75	658.89	P	861.43	843.41	844.40	431.21	8
12	60.04	1403.81	1385.80	1386.78	702.41	S	764.37	746.36	747.34	382.69	7
13	72.08	1502.88	1484.87	1485.85	751.94	V	677.34	659.33	660.31	339.17	6
14	30.03	1559.90	1541.89	1542.87	780.45	G	578.27	560.26	561.25	289.64	5
15	60.04	1646.93	1628.92	1629.91	823.97	S	521.25	503.24	504.22	261.13	4
16	72.08	1746.00	1727.99	1728.97	873.50	V	434.22	416.21	417.19	217.61	3
17	133.04	1906.03	1888.02	1889.00	953.52	C(+57.02)	335.15	317.14	318.12	168.07	2
18	129.11					R	175.12	157.10	158.09	88.06	1

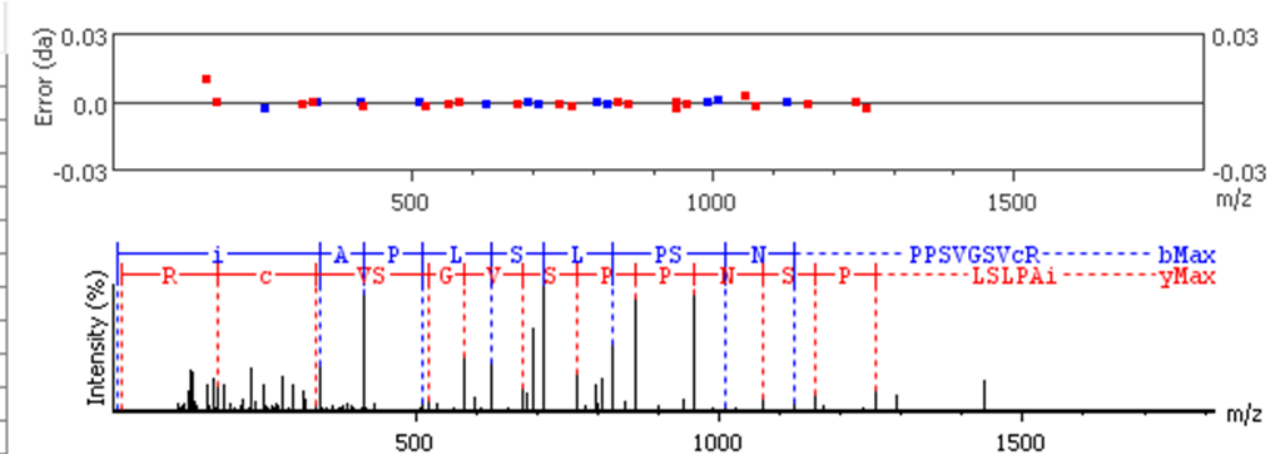

Figure S9. MS1 and MS2 spectra of Ser[136] → Asn (+27.01 Da) residue substitution in gyroxin digested peptide IAPLSLPSNPPSVGSVCR (nonmutated peptide: IAPLSLPSSPPSVGSVCR). Peptide observed at m/z 694.05^{+3}. Fragment ions assigned using PEAKS Studio X+ (BSI).

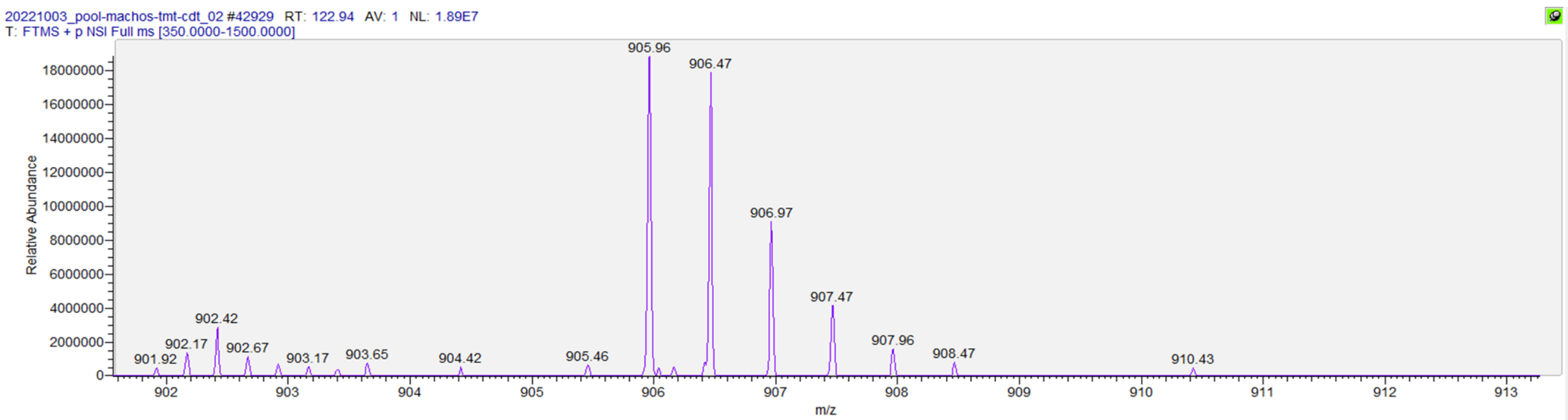

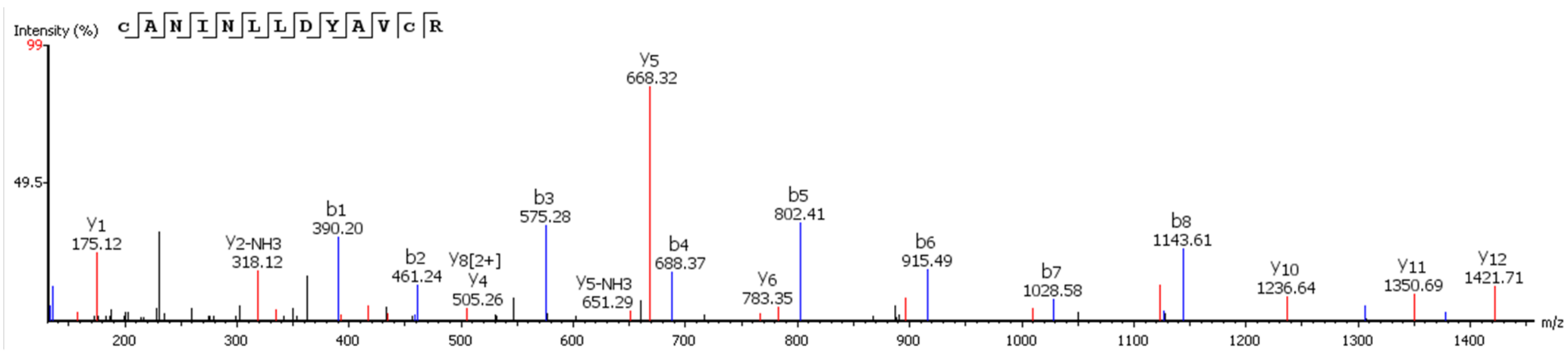

Ion Match | Survey

#	Immonium	b	b-H2O	b-NH3	b (2+)	Seq	y	y-H2O	y-NH3	y (2+)	#
1	362.21	390.20	372.19	373.17	195.60	C(+286.18)					13
2	44.05	461.24	443.23	444.21	231.12	A	1421.71	1403.71	1404.69	711.36	12
3	87.06	575.28	557.27	558.25	288.14	N	1350.69	1332.67	1333.66	675.84	11
4	86.10	688.37	670.35	671.34	344.68	I	1236.64	1218.63	1219.61	618.82	10
5	87.06	802.41	784.40	785.38	401.70	N	1123.56	1105.55	1106.53	562.28	9
6	86.10	915.49	897.48	898.47	458.25	L	1009.51	991.50	992.49	505.26	8
7	86.10	1028.58	1010.57	1011.55	514.79	L	896.43	878.42	879.40	448.71	7
8	88.04	1143.61	1125.59	1126.57	572.30	D	783.35	765.33	766.32	392.18	6
9	136.08	1306.67	1288.66	1289.64	653.83	Y	668.32	650.31	651.29	334.66	5
10	44.05	1377.70	1359.69	1360.68	689.35	A	505.26	487.24	488.23	253.13	4
11	72.08	1476.77	1458.76	1459.75	738.89	V	434.22	416.21	417.19	217.61	3
12	133.04	1636.80	1618.79	1619.78	818.90	C(+57.02)	335.15	317.14	318.12	168.07	2
13	129.11					R	175.12	157.11	158.09	88.06	1

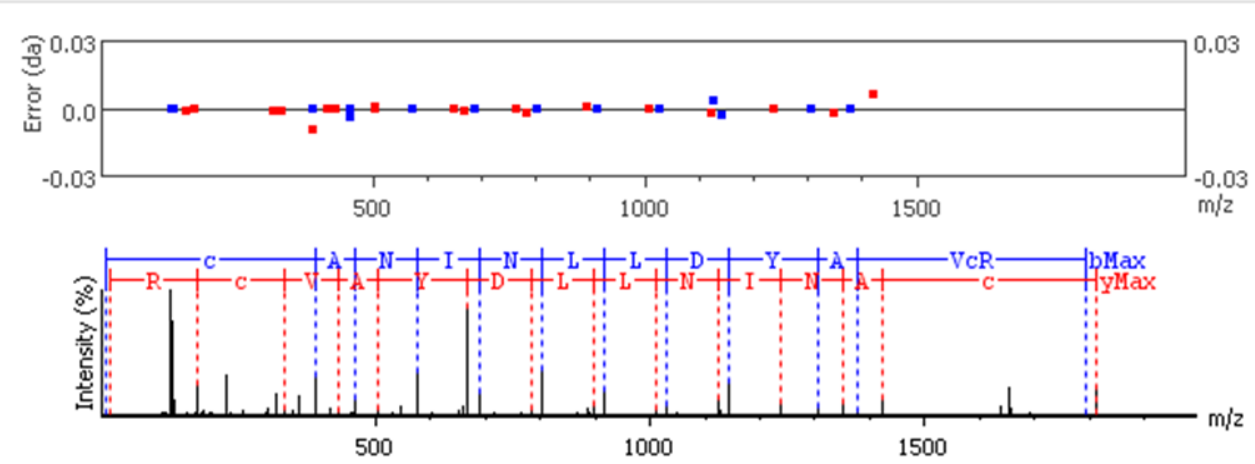

Figure S10. MS1 and MS2 spectra of Glu[174] → Ala (-58.01 Da) residue substitution in gyroxin digested peptide CANINLLDYAVCR (nonmutated peptide: CANINLLDYEVCR). Peptide observed at m/z 905.96^{+2}. Fragment ions assigned using PEAKS Studio X+ (BSI).

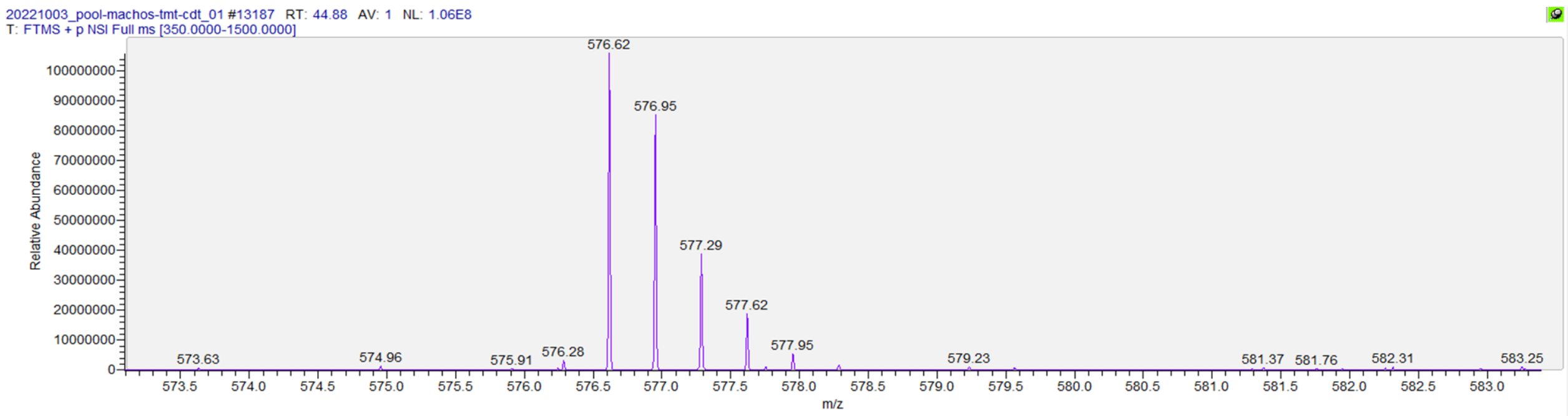

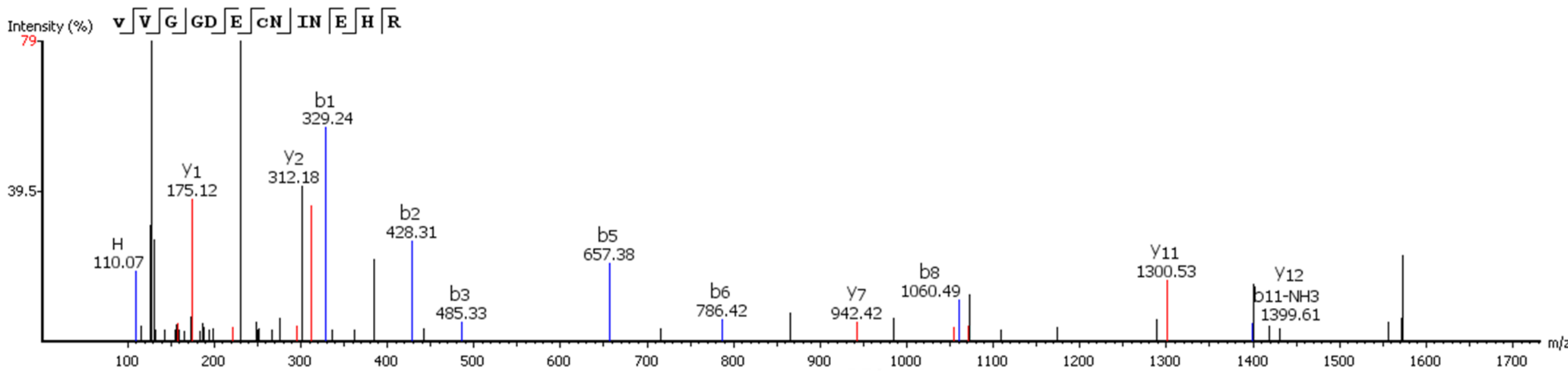

Ion Match

#	Immonium	b	b-H2O	b-NH3	b (2+)	Seq	y	y-H2O	y-NH3	y (2+)	#
1	301.24	329.24	311.23	312.21	165.12	V(+229.16)					13
2	72.08	428.31	410.30	411.28	214.65	V	1399.61	1381.59	1382.57	700.30	12
3	30.03	485.33	467.32	468.30	243.16	G	1300.53	1282.52	1283.51	650.77	11
4	30.03	542.35	524.34	525.32	271.68	G	1243.51	1225.50	1226.48	622.26	10
5	88.04	657.38	639.37	640.35	329.19	D	1186.49	1168.48	1169.46	593.75	9
6	102.06	786.42	768.41	769.39	393.71	E	1071.47	1053.45	1054.44	536.23	8
7	133.04	946.45	928.44	929.42	473.73	C(+57.02)	942.42	924.41	925.39	471.71	7
8	87.06	1060.49	1042.48	1043.47	530.75	N	782.39	764.38	765.36	391.70	6
9	86.10	1173.58	1155.57	1156.55	587.29	I	668.35	650.34	651.32	334.67	5
10	87.06	1287.62	1269.61	1270.59	644.31	N	555.26	537.25	538.24	278.13	4
11	102.06	1416.66	1398.65	1399.61	708.83	E	441.22	423.21	424.19	221.10	3
12	110.07	1553.72	1535.71	1536.70	777.36	H	312.18	294.17	295.15	156.59	2
13	129.11					R	175.12	157.11	158.09	88.06	1

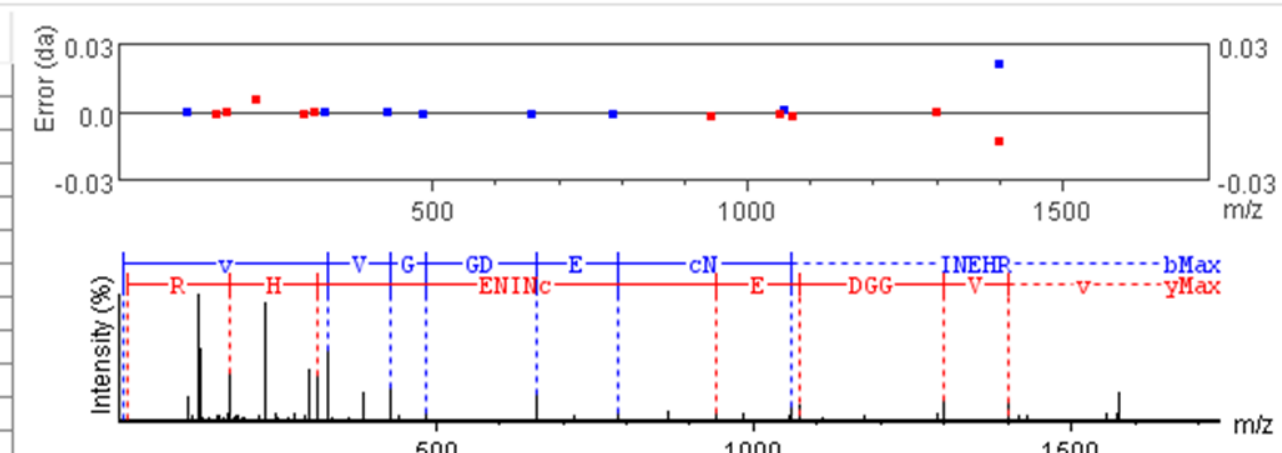

Figure S11. MS1 and MS2 spectra of Ile^{26} → Val (-14.02 Da) residue substitution in gyroxin digested peptide VVGGDECNINEHR (nonmutated peptide: VIGGDECNINEHR). Peptide observed at m/z 576.62^{+3}. Fragment ions assigned using PEAKS Studio X+ (BSI).

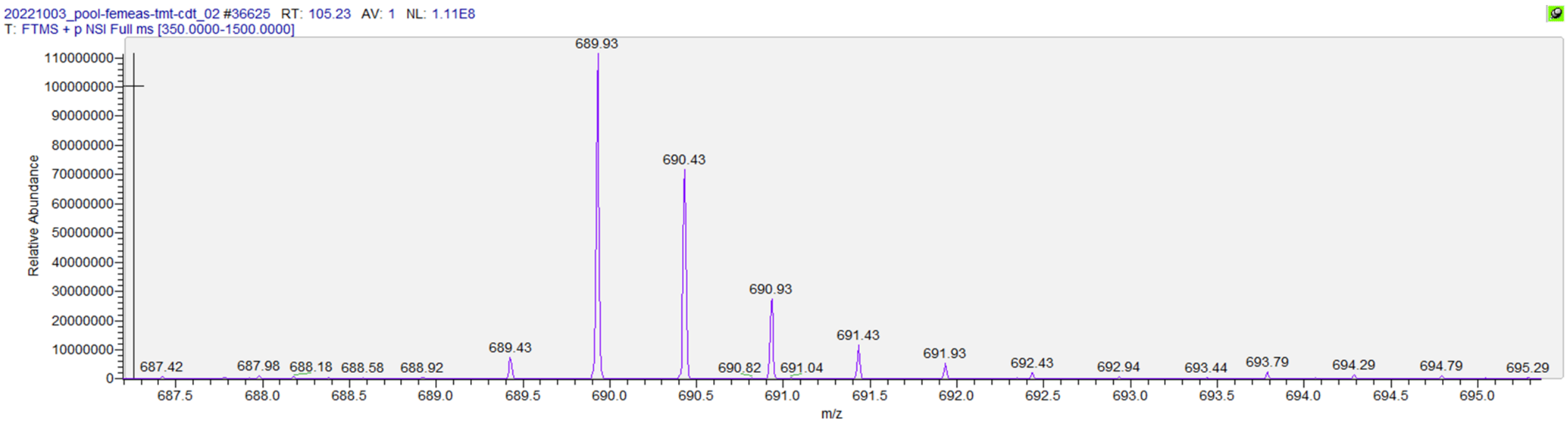

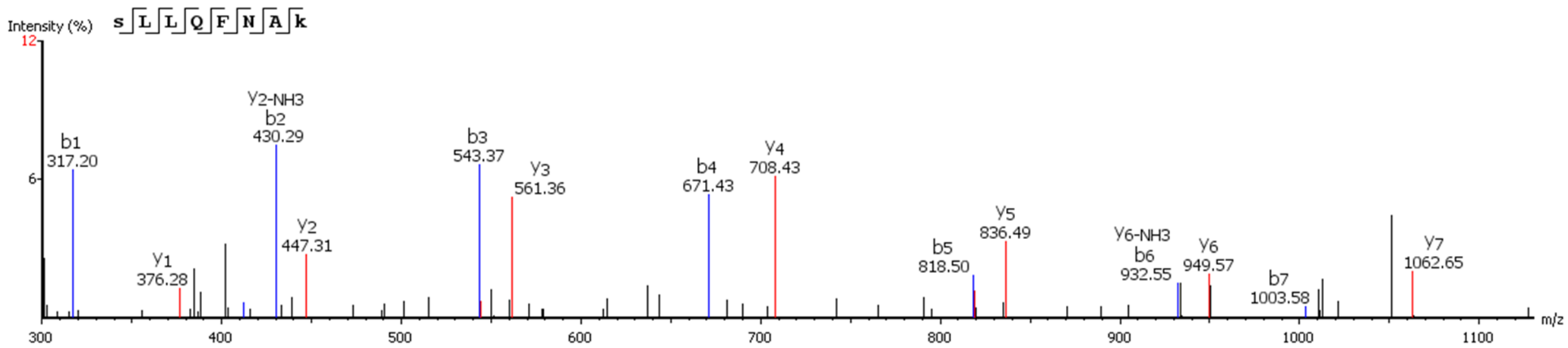

Ion Match | Survey

#	Immonium	b	b-H2O	b-NH3	b (2+)	Seq	y	y-H2O	y-NH3	y (2+)	#
1	289.21	317.20	299.19	300.18	159.09	S(+229.16)					8
2	86.10	430.29	412.28	413.26	215.64	L	1062.65	1044.64	1045.62	531.83	7
3	86.10	543.37	525.36	526.34	272.19	L	949.57	931.56	932.55	475.28	6
4	101.07	671.43	653.42	654.40	336.21	Q	836.49	818.47	819.46	418.74	5
5	120.08	818.50	800.49	801.47	409.75	F	708.43	690.41	691.40	354.71	4
6	87.06	932.55	914.53	915.51	466.77	N	561.36	543.35	544.33	281.18	3
7	44.05	1003.58	985.57	986.55	502.29	A	447.31	429.30	430.29	224.16	2
8	330.27					K(+229.16)	376.28	358.27	359.25	188.64	1

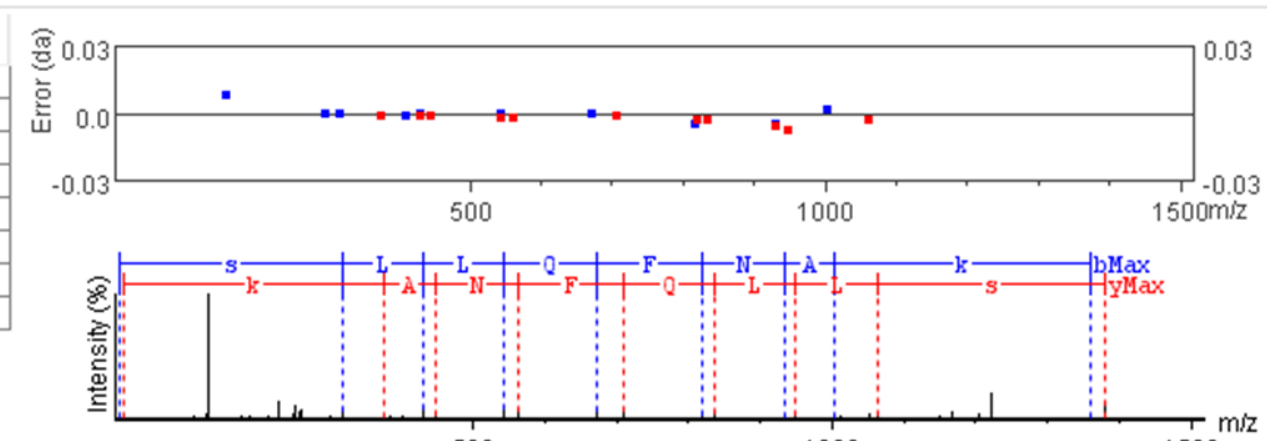

Figure S12. MS1 and MS2 spectra of alanine insertion → $Ala^{23}_{(Insertion)}$ (+71.04 Da) in PLA2 CB subunit digested peptide SLLQFNAK (nonmutated peptide: SLLQFNK). Peptide observed at m/z 689.93^{+2}. Fragment ions assigned using PEAKS Studio X+ (BSI).

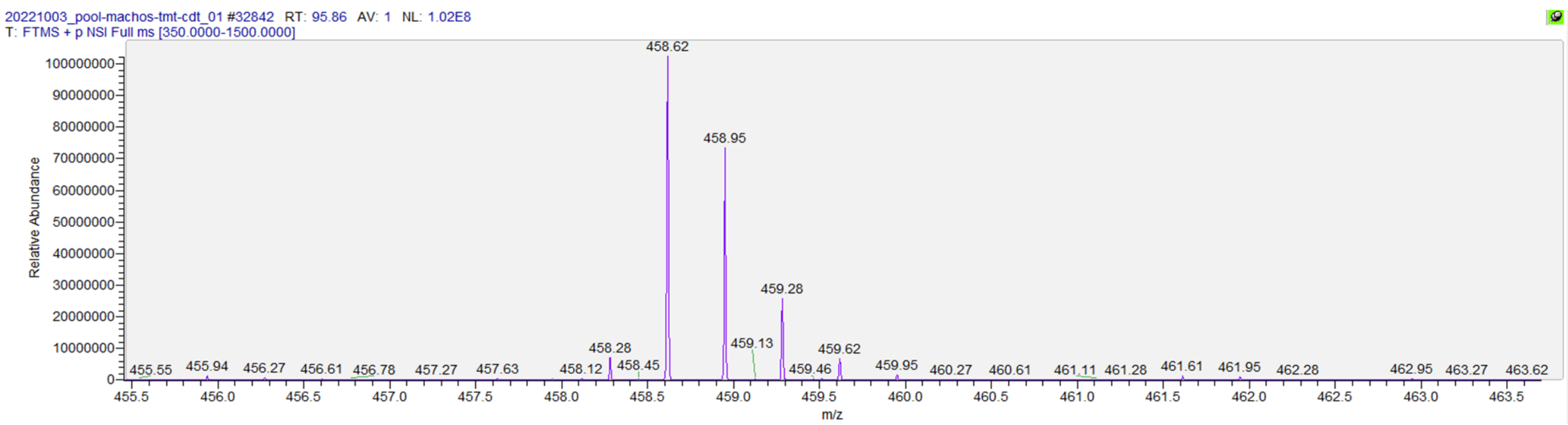

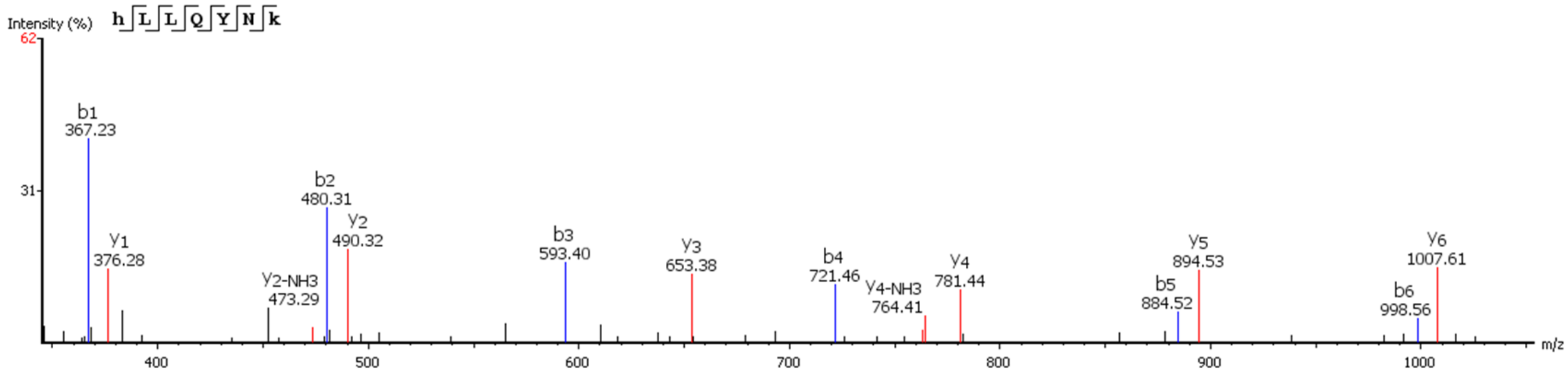

Ion Match

#	Immonium	b	b-H2O	b-NH3	b (2+)	Seq	y	y-H2O	y-NH3	y (2+)	#
1	339.23	367.23	349.22	350.20	184.11	H(+229.16)					7
2	86.10	480.31	462.30	463.29	240.66	L	1007.61	989.60	990.58	504.30	6
3	86.10	593.40	575.39	576.37	297.20	L	894.53	876.51	877.50	447.76	5
4	101.07	721.46	703.45	704.43	361.23	Q	781.44	763.43	764.41	391.22	4
5	136.08	884.52	866.51	867.49	442.76	Y	653.38	635.37	636.35	327.19	3
6	87.06	998.56	980.55	981.54	499.78	N	490.32	472.31	473.29	245.66	2
7	330.27					K(+229.16)	376.28	358.27	359.25	188.64	1

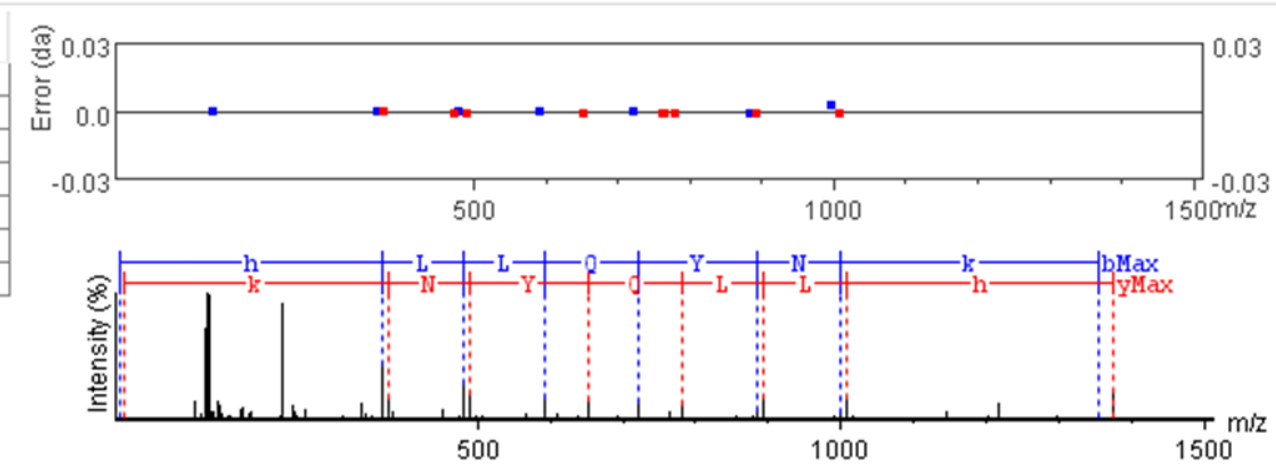

Figure S13. MS1 and MS2 spectra of Phe[21] → Tyr (+15.99 Da) residue substitution in PLA2 CB subunit digested peptide HLLQYNK (nonmutated peptide: HLLQFNK). Peptide observed at m/z 458.62+3. Fragment ions assigned using PEAKS Studio X+ (BSI).

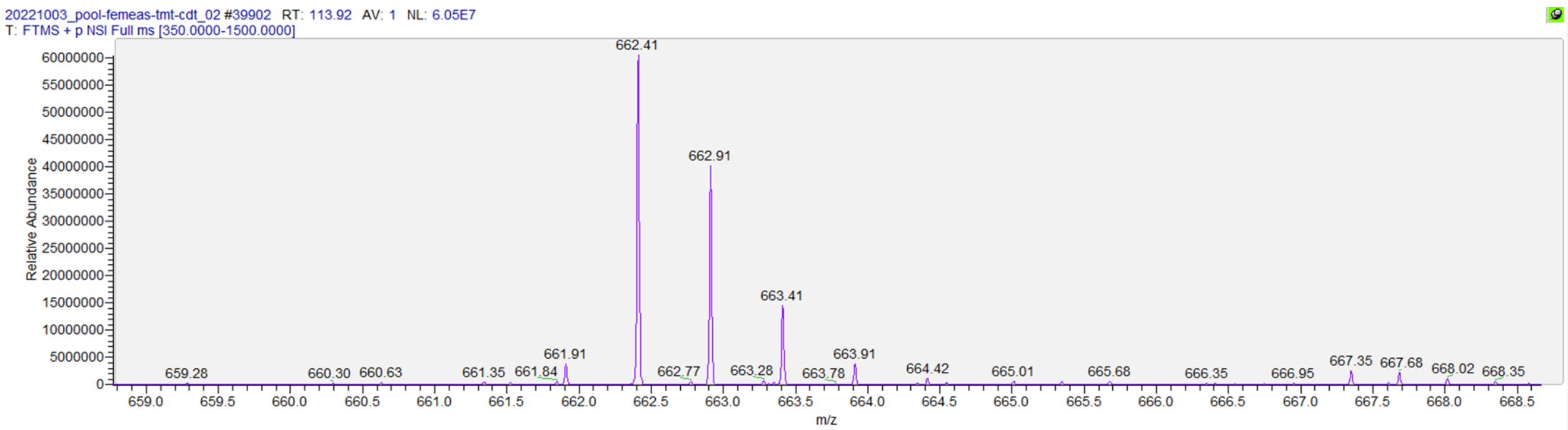

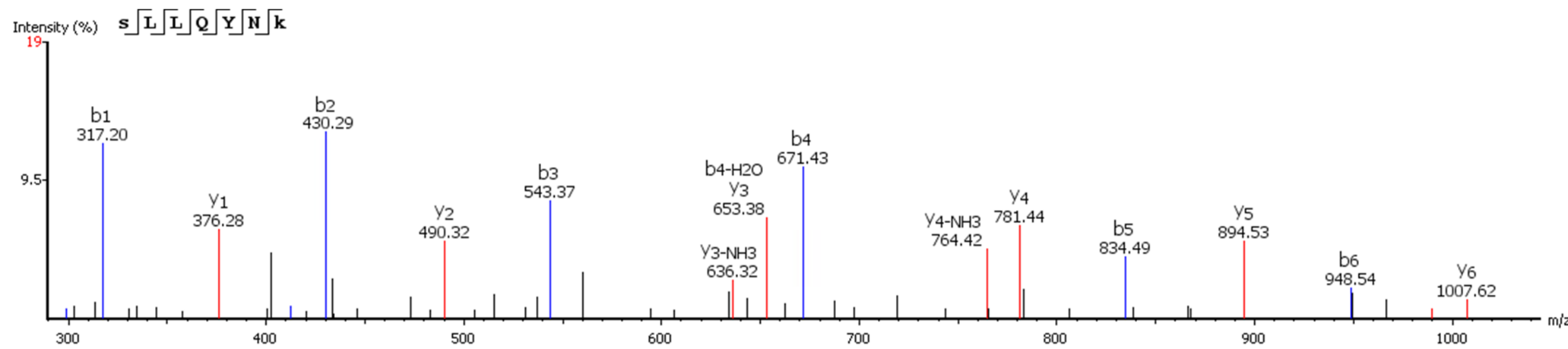

Ion Match | Survey

#	Immonium	b	b-H2O	b-NH3	b (2+)	Seq	y	y-H2O	y-NH3	y (2+)	#
1	289.21	317.20	299.19	300.18	159.10	S(+229.16)					7
2	86.10	430.29	412.28	413.26	215.64	L	1007.62	989.60	990.58	504.30	6
3	86.10	543.37	525.36	526.34	272.19	L	894.53	876.51	877.50	447.76	5
4	101.07	671.43	653.42	654.40	336.21	Q	781.44	763.43	764.42	391.22	4
5	136.08	834.49	816.48	817.47	417.75	Y	653.38	635.37	636.36	327.19	3
6	87.06	948.54	930.53	931.51	474.77	N	490.32	472.31	473.29	245.66	2
7	330.27					K(+229.16)	376.28	358.27	359.25	188.64	1

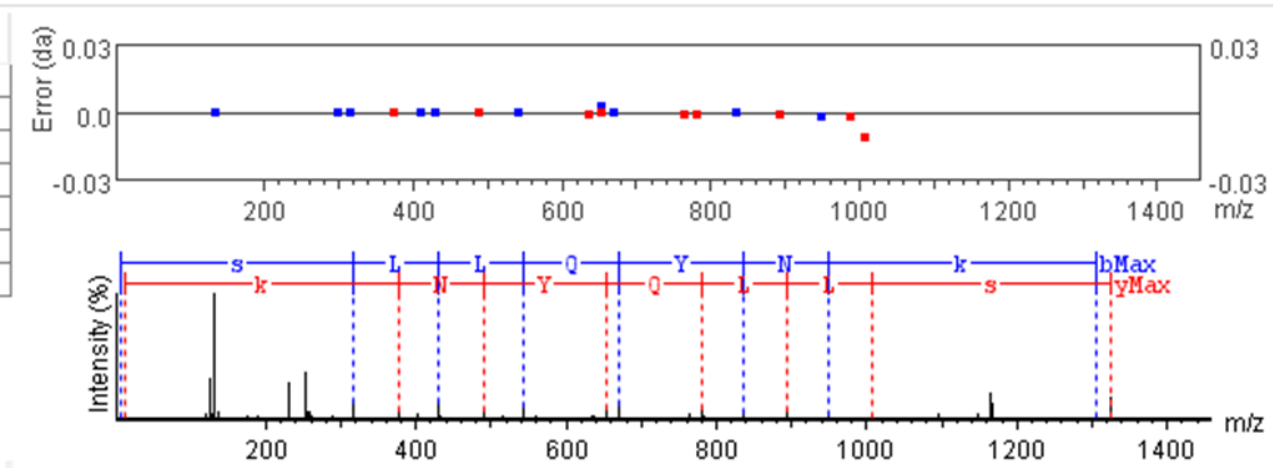

Figure S14. MS1 and MS2 spectra of Phe^{21} → Tyr (+15.99 Da) residue substitution in PLA2 CB subunit digested peptide SLLQYNK (nonmutated peptide: SLLQFNK). Peptide observed at m/z 662.41^{+2}. Fragment ions assigned using PEAKS Studio X+ (BSI).

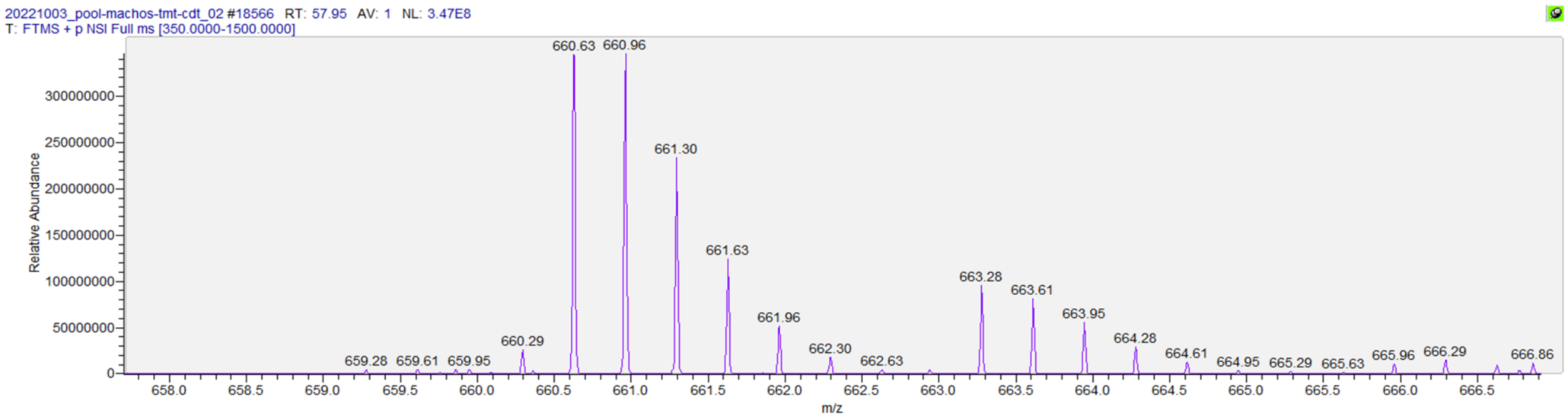

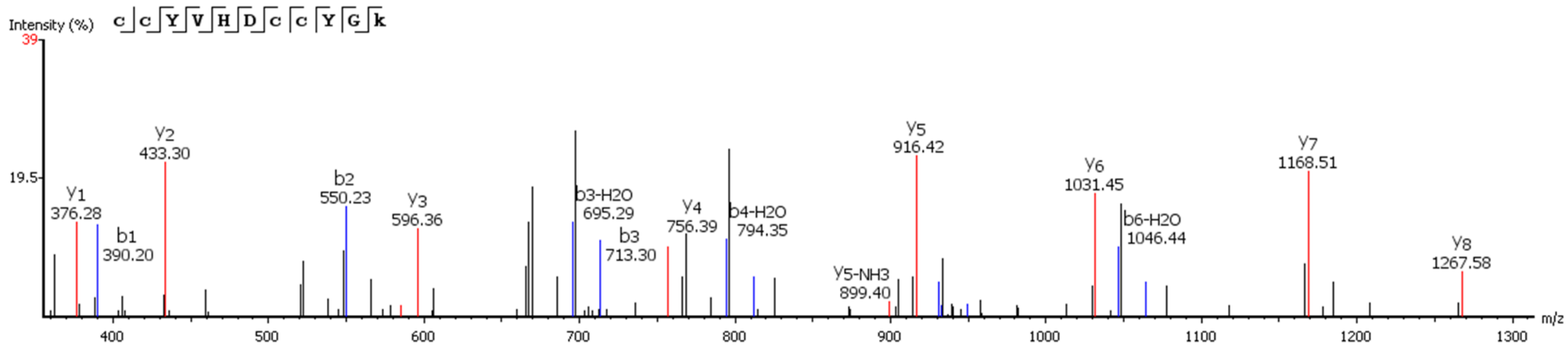

Ion Match | Survey

#	Immonium	b	b-H2O	b-NH3	b (2+)	Seq	y	y-H2O	y-NH3	y (2+)	#
1	362.21	390.20	372.19	373.17	195.60	C(+286.18)					11
2	133.04	550.23	532.22	533.21	275.62	C(+57.02)	1590.67	1572.66	1573.64	795.84	10
3	136.08	713.30	695.29	696.27	357.15	Y	1430.63	1412.63	1413.61	715.82	9
4	72.08	812.36	794.35	795.34	406.68	V	1267.58	1249.57	1250.55	634.29	8
5	110.07	949.42	931.41	932.42	475.21	H	1168.51	1150.50	1151.48	584.76	7
6	88.04	1064.45	1046.44	1047.42	532.72	D	1031.45	1013.44	1014.42	516.22	6
7	133.04	1224.48	1206.47	1207.45	612.74	C(+57.02)	916.42	898.41	899.40	458.71	5
8	133.04	1384.51	1366.50	1367.48	692.76	C(+57.02)	756.39	738.38	739.36	378.70	4
9	136.08	1547.57	1529.56	1530.55	774.29	Y	596.36	578.35	579.33	298.68	3
10	30.03	1604.60	1586.59	1587.57	802.80	G	433.30	415.29	416.27	217.15	2
11	330.27					K(+229.16)	376.28	358.27	359.25	188.64	1

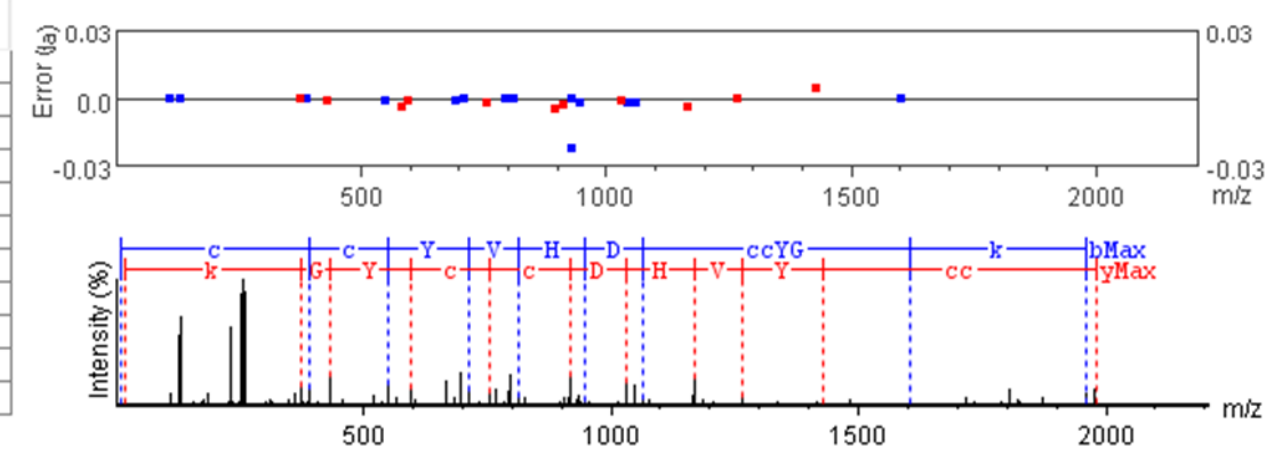

Figure S15. MS1 and MS2 spectra of Phe[61] → Tyr (+15.99 Da) residue substitution in PLA2 CB subunit digested peptide CCYVHDCCYGK (nonmutated peptide: CCFVHDCCYGK). Peptide observed at m/z 660.63^{+3}. Fragment ions assigned using PEAKS Studio X+ (BSI).
